# Characterisation and calibration of low-cost PM sensors at high temporal resolution to reference-grade performance

**DOI:** 10.1016/j.heliyon.2023.e15943

**Published:** 2023-04-29

**Authors:** Florentin M.J. Bulot, Steven J. Ossont, Andrew K.R. Morris, Philip J. Basford, Natasha H.C. Easton, Hazel L. Mitchell, Gavin L. Foster, Simon J. Cox, Matthew Loxham

**Affiliations:** a Faculty of Engineering and Physical Sciences, University of Southampton, Southampton, UK; b Southampton Marine and Maritime Institute, University of Southampton, Southampton, UK; c BizData, 278 Collins St, Melbourne, VIC, 3000, Australia; d National Oceanography Centre, Southampton, UK; e Faculty of Environmental and Life Sciences, University of Southampton, Southampton, UK; f School of Ocean and Earth Science, National Oceanography Centre, University of Southampton, UK; g School of Clinical and Experimental Sciences, Faculty of Medicine, University of Southampton, Southampton, UK; h National Institute for Health Research Southampton Biomedical Research Centre, Southampton, UK; i Institute for Life Sciences, University of Southampton, Southampton, UK

**Keywords:** Low-cost sensors, Calibation, Air pollution, Particulate matter, PM2.5, Machine learning

## Abstract

Particulate Matter (PM) low-cost sensors (LCS) present a cost-effective opportunity to improve the spatiotemporal resolution of airborne PM data. Previous studies focused on PM-LCS-reported hourly data and identified, without fully addressing, their limitations. However, PM-LCS provide measurements at finer temporal resolutions. Furthermore, government bodies have developed certifications to accompany new uses of these sensors, but these certifications have shortcomings. To address these knowledge gaps, PM-LCS of two models, 8 Sensirion SPS30 and 8 Plantower PMS5003, were collocated for one year with a Fidas 200S, MCERTS-certified PM monitor and were characterised at 2 min resolution, enabling replication of certification processes, and highlighting their limitations and improvements. Robust linear models using sensor-reported particle number concentrations and relative humidity, coupled with 2-week biannual calibration campaigns, achieved reference-grade performance, at median PM_2.5_ background concentration of 5.5 μg/m^3^, demonstrating that, with careful calibration, PM-LCS may cost-effectively supplement reference equipment in multi-nodes networks with fine spatiotemporality.

## Highlights

• Low-cost PM sensors are seldom studied at the full extent of their temporal resolution and require efficient data quality processes to reach a low operational cost.
• This study collocated 16 low-cost PM sensors with a reference instrument for a yearlong study at a fine temporal resolution.
• Different calibration methods, frequencies and duration were tested at 2 min resolution.
• The setup enabled the replication of sensor performance certification processes and highlighted improvements.
• Carefully selected calibration strategies, based on sensor-reported particle number concentration, enabled low-cost PM sensors to reach reference-grade performances at 2 min resolution.

## 1 Introduction

Air quality varies with fine spatiotemporal granularity and can have heterogeneous composition and concentration over a single urban area [1]. Current air quality monitoring stations operate legally-certified equipment to measure Particulate Matter (PM) mass, to verify compliance with PM concentration limits. However, they are expensive to acquire, run and maintain. This limits the number of sampling locations and the spatial resolution of monitoring (e.g. the city of Southampton in the UK, with a footprint of 52 km^2^ only has one monitoring station for PM_2.5_). Additionally, reference instruments are certified for daily averages and generally provide data at an hourly resolution. Low-cost PM sensors, which usually determine PM mass concentration through the extent to which PM scatter light, are much less expensive (£10-£100s, vs £10k-£100k for reference equipment), are small, require substantially less power, and are less demanding of expert maintenance, and thus are more logistically and financially suitable for installation on a wider scale and they provide measurements every second. These sensors have the potential to address both the spatial and temporal granularity required to better our understanding of fine air pollution variations.

In the UK, the Environment Agency developed certification standards for PM measurement instruments, known as the Monitoring Certification Schemes (MCERTS) for UK PM [2] based on the European Directive 2008/50/EC which sets Data Quality Objective for air pollution measurement equipment to be legally binding [3]. The MCERTS for Indicative Ambient Particulate Monitors [4], is a variation of the MCERTS for UK PM that was developed with lower Data Quality Objectives to certify instruments that can provide information about air quality without being legally binding. It requires testing a pair of PM measurement instruments on at least 40 pairs of 24 h data points and requires that the sensors obtain an expanded uncertainty below 50% (compared to 25% for MCERTS for UK PM) and a between unit uncertainty <5 μg/m^3^ (compared to 2.5 μg/m^3^ for MCERTS for UK PM) for the full dataset and for the two subsets of data obtained by splitting the data between concentrations greater and smaller than 18 μg/m^3^. The expanded uncertainty and the between unit uncertainty are described in the methods section of this paper. The certification also states that the certified instruments can then be used for quantitative measurements provided they have been calibrated through a two-week collocation with a reference instrument at the location they are installed, and that re-calibration is required each year or less often. However, this certification protocol does not require a verification of the performances of sensors at finer temporal resolution while most modern instruments, and especially instruments based on optical measurement techniques, can provide measurement down to 1 s resolution. This represents a missed opportunity to characterise the sensors at time scale that would benefit our understanding of personal exposure to PM.

There has been a number of studies in recent years about the limitations of low-cost PM sensors [[5], [6], [7]], with an emerging consensus that they are suitable for understanding PM concentrations as long as the data is carefully interpreted [8]. Several studies have shown that agreement between sensors and reference instruments could be improved through field calibration via techniques including machine learning or multilinear regression [9,10]. Two main approaches have been explored in the literature to correct and calibrate low-cost PM sensors: (i) by correcting physical properties of the particles and (ii) through an empirical approach. Furthermore, two correction methods are used in the literature for the physical approach. The first corrects for the hygroscopic growth of particles using the *κ*-koehler theory and the second method is based on Laulainen correction [11].

For the empirical approaches, a range of methods are used: Generalised Additive Models (GAM) [[12], [13], [14], [15], [16]], linear models [[17], [18], [19], [20], [21], [22], [23], [24]], and Machine Learning (Artificial Neural Network (ANN) [18,24], Random Forest (RF) [16,19,24], k-Nearest-Neighbour (KNN) [18,24], Gradient Boosting Regression Tree (GBRT) [17,24], eXtreme Gradient Boosting [23], Feedforward Neural Network [23,25], and Support Vector Machine Regression [25]). Lee *et al.* [26] compared twelve calibration methods, including some of the calibration methods above-mentioned, at 5 min averaging on three Plantower PMS7003 against a Beta Attenuation Monitor (BAM) for 7.5 months and obtained very similar results for most of the calibration methods tested. Malings *et al.* [15] compared the physical approach based on the *k*-Koehler theory and some empirical methods on nine Purple Air PA-II (each containing two Plantower PMS5003) and obtained similar results for both approaches. The two studies together indicate that different calibration methods apparently yield similar results; therefore, improvements to the performances of the sensors may be better focused on improving calibration strategies or on using different combinations of environmental and physical variables. For instance, Zusman *et al.* [14] is the only study, to the best of our knowledge, that used the particle number concentrations reported by the sensors to calibrate them at an hourly resolution. This was done through a GAM on daily data for the Plantower PMSA003, and they obtained a cross-validated R^2^ of 0.96 and a Root Mean Square Error (RMSE) of 1.15 μg/m^3^. Additionally, while there is interest in fine temporal resolution data, only a few studies considered sampling averaging <1 h for the calibration [18,20,27,28] and calibrating sensors at higher time frequencies may reveal additional issues that are not encountered with longer time averaging windows. Therefore, this paper not only explores different calibration techniques but also different calibration variables (such as PM concentrations and particle number concentrations), calibration frequencies and durations at a high temporal resolution.

In this study, eight of each of the Sensirion SPS30 and the Plantower PMS5003 models were deployed from July 2020 to July 2021 outside the National Oceanography Centre, Southampton, along with a Fidas 200S, a reference-grade instrument, MCERTS for UK Particulate Matter [2] certified for PM monitoring, using the low-cost air quality monitors developed by Johnston *et al.* [29] These air quality monitors have been validated through previous studies along with the response of the sensors to different environmental parameters [5,6]. The sensors and the Fidas collected data every 10 s.

The objectives of this study are to: (1) characterise the behaviour of low-cost PM sensors at 10 s resolution in real-world conditions; (2) determine a rationale for choosing a particular averaging time for low-cost PM sensors; (3) provide recommendations for a potential certification of low-cost PM sensors at these temporal resolutions; (4) compare and evaluate calibration methods for the sensors; (5) characterise the evolution of the performances of the calibrated sensors through time; (6) compare different calibration scenarios to provide recommendations about how to calibrate the sensors to obtain the best performance possible.

## 2 Materials and methods

### 2.1 Experimental design

The collocation was begun on March 16, 2020 at Empress dock at the National Oceanography Centre, Southampton. The location of the study is presented in Supplementary Figure S2. Southampton is a coastal city with a major goods and cruise port, the city include several industries, a high-way, an airport.

**Fig. 1:**
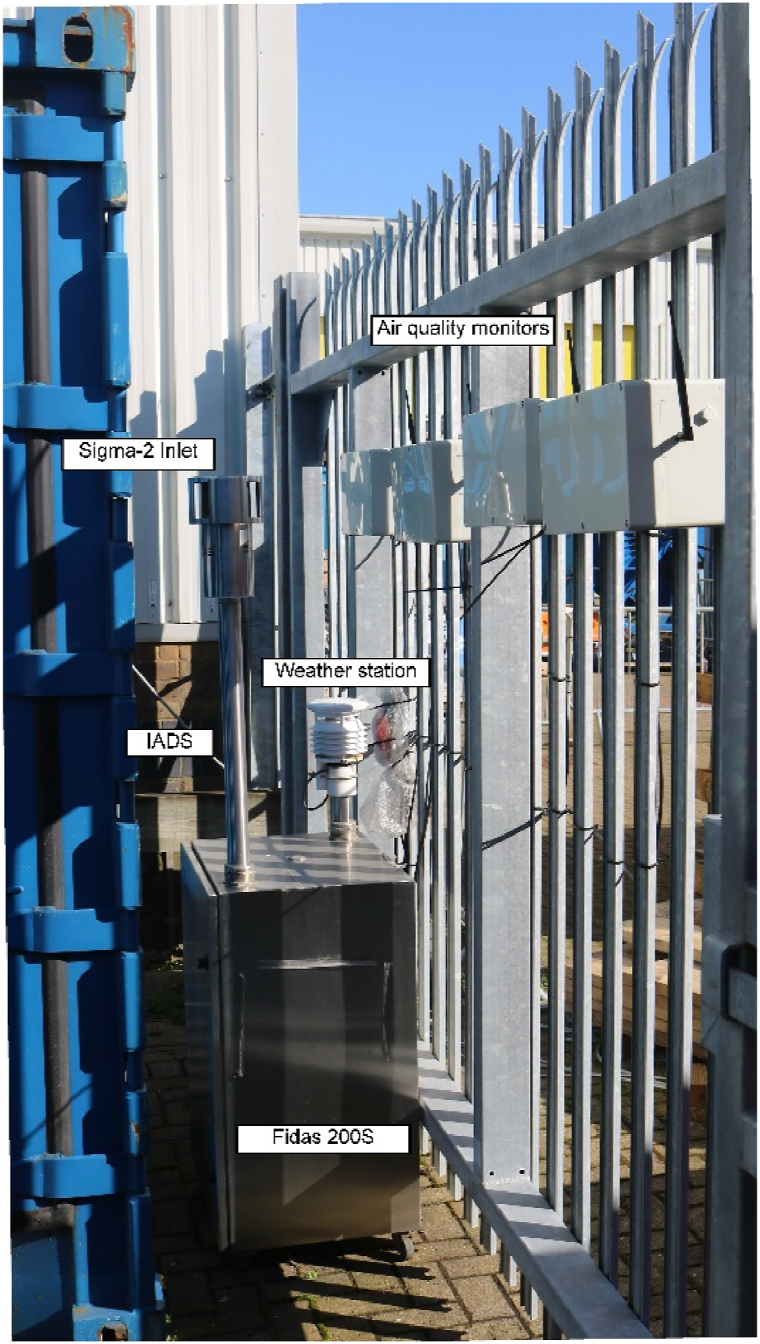
Picture of the deployment set up, at the docks of the National Oceanography Center in Southampton, UK. The Fidas 200S is the grey enclosure on the left of the picture topped by its Sigma-2 inlet on the left and its weather station on the right. The air quality monitors are the white boxes mounted onto the fence on the right of the picture.

Four air quality monitors were used for the study, each containing a duplicate of five sensor models for a total of 40 low-cost PM sensors, similarly to Bulot *et al.* [6], although this study focuses on 16 of these sensors: eight Plantower PMS5003 and eight Sensirion SPS30. The inlet of the air quality monitors was positioned at the same height as the inlet of the Fidas 200S and the enclosure were secured to a fence against which the Fidas 200S was positioned. Fig. 1 shows the deployment setup. The instruments were installed at the corner of the stores of the National Oceanography Centre, facing the manipulation area of the docks of the National Oceanography Centre. The air quality monitors were mounted on a fence, attached to the stores, which was kept closed for the duration of the study. The Fidas 200S was installed on the other side of the fence, between a container and the fence, to protect it against damage or tampering. The inlet of the sensors and the Fidas 200S are located less than 50 cm away from each other. Initially, there was no network connectivity, and due to the start of the first UK COVID-19 lockdown shortly after the deployment, the setup could not be accessed before the end of June 2020. The issue was resolved by installing a modem with a SIM card to gain remote access to the equipment and be able to download the data remotely. Therefore, the data presented here ranges from July 01, 2020 to July 03, 2021. The Fidas 200S was set to store data at 1 s resolution and its limited internal memory does not allow to store more than 10 days of data at this temporal resolution. The air quality monitors were powered through Power over Ethernet (PoE) and the Fidas 200S was powered on mains.

**Fig. 2:**
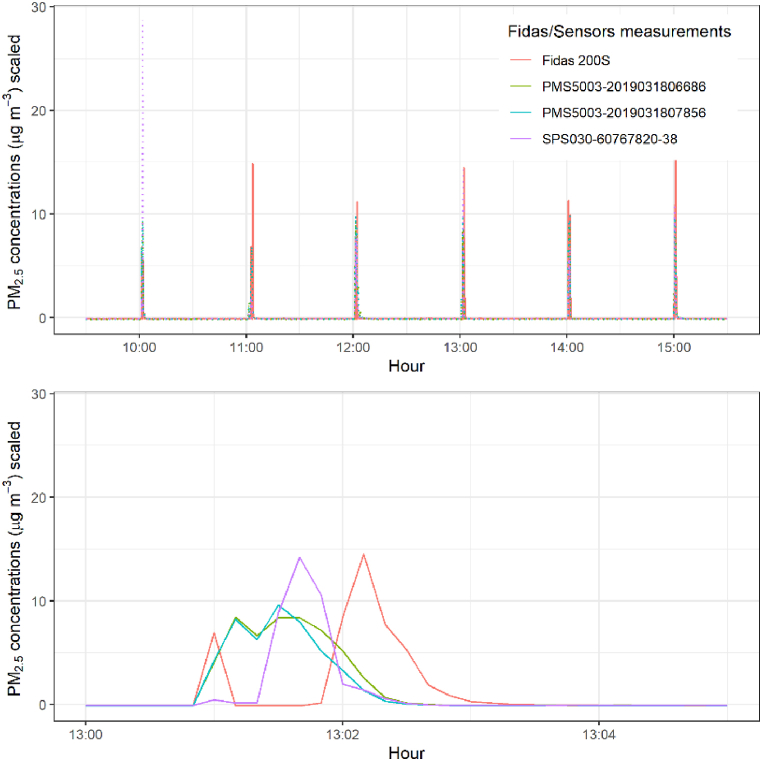
Peaks of PM generated by combustion of incense sticks for 30 s every hour. The upper panel presents an overview of the peaks to give an indication about the timing of the six peaks generated, and the lower panel magnifies the peak generated at 13.00. PM concentrations reported by the three sensors considered and the Fidas 200S were scaled for comparison. This peak is representative of the other peaks measured.

Supplementary Fig. S3 and S4 show the time series of the RH, temperature and PM_2.5_ concentrations recorded by the Fidas 200S during the period of the study at 2 min averaging. The periods without datapoints are the periods when no data was available from the Fidas 200S, mostly due to network and storage issues. These periods are not included in the rest of this study. During this period, the RH ranged from 24% to 100% with a median value of 82%, a 25th percentile of 70% and a 75th percentile of 91%. Temperature ranged between 2 °C and 34 °C with a median value of 15 °C, a 25th percentile of 12 °C and a 75th percentile of 18 °C. PM_2.5_ concentrations ranged between 0.4 μg/m^3^ and 70.2 μg/m^3^ with a median value of 5.5 μg/m^3^, a 25th percentile of 3.8 μg/m^3^ and a 75th percentile of 8.7 μg/m^3^. It is important to highlight that as this deployment was undertaken during the COVID-19 pandemic in 2020 and 2021, during lockdowns in the UK, PM concentrations and composition patterns may notably differ from previous years. Supplementary Figure S5 shows the time series of the monthly averaged PM_2.5_ concentrations measured by the AURN station Southampton Centre (located on the map in Supplementary Figure S1). The monthly concentration patterns and average concentrations during the study are not sensibly different from other periods.

During the early stages of the analysis, a delay between the peaks recorded by the Fidas 200S and the sensors at 10 s resolution was noticed. To characterise this delay, on the November 03, 2020, incense sticks were simultaneously exposed to the Sigma-2 sampling head of the Fidas 200S and below one of the sensor boxes, for 30 s every hour between 10.00 and 15.00. The data corresponding to this specific investigation was not included for the rest of the analysis but was used to characterise this observed delay between sensing systems.

The RH measured by the air quality monitors using the Sensirion SHT35 sensors was compared with the RH measured by the weather station of the Fidas 200S–WS300-UMB weather station. The RH from the air quality monitors had a negative relative bias of 15% compared to the RH reported by the Fidas 200S meaning that the reported RH within the enclosure of the sensors was consistently lower than the RH outside, assuming the RH from the Fidas 200S was accurate. For temperature, the air quality monitors had positive relative bias of 18%, suggesting the temperature inside the enclosure was consistently higher than the outdoor temperature. These biases did not vary significantly with time. These biases were assumed to result from the residual heat generated by the sensors and the electronics hosted in the air quality monitor enclosure. For the analysis, the RH reported by the Fidas 200S was used.

### 2.2 Instruments used

#### 2.2.1 Fidas 200S

The Fidas 200S (Palas GmbH, Karlsruhe, Germany) is an optical instrument that meets the criteria of equivalence for Monitoring of Particulate Matter and it is certified by the Environment Agency's MCERTS for UK Particulate Matter and MCERTS for Continuous Ambient Air Monitoring System. It is an aerosol spectrometer, based on light-scattering, which can detect particles from 0.18 to 18 μm diameter. It outputs PM mass concentration and particle size distribution every second and has several data formats available. It had been calibrated every three months during the study as per its requirements to check a number of calibration parameters. It is equipped with an Intelligent Aerosol Drying System (IADS), which is controlled by ambient air temperature, humidity and pressure, to dry the flow of air sampled. These environmental parameters were monitored and recorded by a built-in WS300-UMB weather station (Lufft, Fellbach, Germany) measuring air temperature (±0.2 °C), RH (±2% RH) and air pressure (±0.5 hPa) every 2 min.

The Fidas 200S has a flow rate of 4.8 L/min and a sampling line 1.4 m long, with the IADS. The time spent by a particle within this heated inlet is estimated to be around 8 s. It is equipped with a Sigma-2 inlet to enable constant sampling no matter the wind conditions. The transit time within the Sigma-2 inlet varies with wind conditions and adds further delay in detection which is hard to estimate as it will (according to personal communications with the supplier) vary with wind conditions. The Fidas 200S samples at 1000 Hz and reports an averaged PM_2.5_ reading every second which is updated every 30 s and subjected to a 15 min right aligned rolling averaging window meaning that each 30 s data point is taken as the mean value of the last 15 min. The readings per second are also available and were used during this study.

#### 2.2.2 PM sensors

This study focuses on two models of PM sensors: the Sensirion SPS30 and the Plantower PMS5003. In Bulot *et al.* [6], the performance of the five models of low-cost sensors when monitoring transient events of PM air pollution was characterised for two combustion sources of PM and variations were observed between the responses of the sensors to two combustion sources. The study used the air quality monitors developed in Johnston et al. [29], presented in Fig. S1B, and out of the five models of low-cost PM sensors tested, the two sensors that performed the best were the Sensirion SPS30 and the Plantower PMS5003 and as such they were chosen as the focus of this study. The sensors and the air quality monitors that host them is presented in Supplementary Figure S1.

The Plantower PMS5003 reports six size bins called gr03μm, gr05μm, gr10μm, gr25μm, gr50μm and gr100μm; gr05μm represents the particle concentration number of particles greater than 0.5 μm and so on. The Sensirion SPS30 reports size bins called n05, n1, n25, n4 and n10 where nXX represents the particle number concentration of particles between 0.3 to X.X μm (i.e. n05 is between 0.3 and 0.5 μm). There is concern in the literature about whether these low-cost PM sensors can accurately segregate the particle number concentration into different size bins [5, [30], [31], [32]]. To verify this, linear models between the different size bins were computed and are presented in Supplementary Tables S1 and S2. For the Sensirion SPS30 the bins n1 to n10 obtained R2 between 0.998 and 1 and slopes between 0.99 and 0.999 between each other, while n05 obtained slightly lower R^2^, although >0.98, and a slope of 0.86–0.87 with the other bins. This suggests that there was no actual differences between the bins apart from n05. As a consequence, and to avoid over–fitting, only bin sizes n05 and n1 were used for the calibration models. According to the manufacturer, n4 and n10 are computed from the other bin sizes of the sensors but not actually measured. For the Plantower PMS5003, the different bins presented much more variability in the R^2^, and the slopes calculated, therefore, all the bins were used for the calibration. Note that this does not denote whether the sensors report bin sizes accurately.

There are generally two types of low-cost PM sensors, integrating nephelometers, that measures the light scattered by an ensemble of particles, and single particle counters (OPC), which are designed to count individual particles. The manufacturers of the models of sensors used here do not specify to which category their sensor belong about which types it belongs. Ouimette *et al.* [30] showed that the Plantower PMS5003 behaved like an integrating nephelometer. In Bulot *et al.* [6], during a laboratory comparison of Plantower PMS5003, Sensirion SPS30 and Alphasense OPC-R1, the Plantower PMS5003 and the Sensirion SPS30 obtained similar results, different from the Alphasense which is an OPC. This lead to suggest that the Sensirion SPS30 utilises a similar measuring mechanism than the Plantower PMS5003. Integrating nephelometers are less susceptible to RH than OPC [31] although they are still impacted. It is therefore important to differentiate between studies on OPCs and studies on integrating nephelometers.

#### 2.2.3 Calibration methods

For the calibration of the low-cost PM sensors with the Fidas 200S, the following variables were used: sensor-reported PM mass concentrations (PMmass), sensor-reported PM particle number concentrations (PMnumber) and RH (measured by the Fidas 200S weather station). Temperature was found to have a R2 of 0.47 with RH during the duration of the study and as such has not been considered as being a potential confounding factor for RH. Hagler *et al.* [32] advise to only consider variables for which a measurement artefact had been demonstrated, including potentially: RH, temperature, elapsed time since manufacture, measurement related to aerosol refractive index, auto-zero data and monitors in close proximity. They list as questionable the following parameters: wind speed and direction; data from neighbouring monitors; local emission information or surrogate for emissions; temporal factors other than elapsed time of use; atmospheric mixing height; location of relative sources. The risk with including the “questionable” variable listed above is to develop a calibration that works under very specific conditions with a potential influence of seasonality. Although it is necessary to weigh cautiously which parameters to include for the calibration, given the dependence on composition observed in Bulot *et al.* [6], it would be useful to include variables such as wind direction and speed, local emission information, atmospheric mixing height and location of relative sources as proxies for particle composition. Another approach is to use some of these variables to define validity boundaries for the calibration methods develop. Lee *et al.* [26] developed such a methodology for which they use a 2-D grid of temperature and RH within each cell of which a calibration method is selected. This is important when developing location specific calibrations. For this study, focusing on improving the performances of the sensors at fine temporal resolutions, these methods and additional variables have not been used.

The calibration methods selected were used by previous studies on low-cost PM sensors at coarser temporal resolution. The objective here is to assess a variety of ready-to-use calibration methods, detailed earlier, and summarised in Supplementary Table S1 including: Linear Regression based on ordinary least square (LR) and based on Orthogonal Least Square (OLS), Multi- Linear Regression (MLR) on PM_*mass*_ including RH and on PM_*number*_ including RH, *κ*-koehler correction on PM_*mass*_, Laulainen correction on PM_*mass*_,Robust Linear Model (RLM) on PM_*mass*_ including RH and on PM_*number*_ including RH. Support Vector Machines (SVM), Gradient Boosting Machines (GBM) on PM_*mass*_ with or without RH were also tested briefly and are described in the Supplementary information Section 3.1. *κ*-koehler correction on the particle number concentration proposed by Di Antonio et al. [27] has not been tested here because of the limited number of particle size bins available with the two models of sensors considered here. According to Malings *et al.* [15], it is possible to use *κ*_*bulk*_ values from neighbouring sites if the information is unavailable locally. Although the limitations of this approach are recognised, *κ*_*bulk*_ = 0*.*16 was used as described in Birmingham by Crilley *et al.* [28] and *ρ*_*p*_ the density of the particles, is taken as 1*.*6 g/cm^3^. For *κ*-koehler and Laulainen corrections, a second stage calibration was applied using LR and OLS. RLM use a M-estimator to reduce the influence of outliers [33]. The different calibration methods used in this study are presented in Supplementary Table S3. They were implemented on PM mass concentration, RH and temperature using the package “MASS” [34] in R. The RLM chosen uses the Huber M-estimator to detect outliers, the Tukey Bi-square and Hampel M-estimators [35] have also been tested briefly and obtained similar results to the Huber M-estimator.

### 2.3 Method selection

To evaluate the impact of different calibration methods, an initial analysis was conducted on the subset of data from July 01, 2020 to August 23, 2020. The first 14 days of this period were used to calibrate the sensors and the 40 remaining days were used to evaluate the calibration. This enabled some of the calibration methods to be discarded given the low performances obtained, which is further detailed in the Supplementary information. Following that, the evolution of the performance of the sensors calibrated using the first two weeks of the dataset were evaluated monthly. To determine robustly the most suitable calibration techniques, in a second analysis, the remaining calibration methods were tested on all possible subsets of the data containing two weeks of calibration and 40 consecutive days of evaluation. This enabled to cross-validate the method selection. Finally, we defined calibration scenarios with different calibration frequencies and durations. These scenarios are evaluated using all the possible subsets of data for cross-validation. The scenarios considered are: (1) one week of calibration followed by eight weeks of deployment and another week of calibration (236 subsets available throughout the study); (2) two weeks of calibration followed by eight weeks of deployment (236 subsets); (3) two weeks of calibration followed by eight weeks of deployment and another two weeks of calibration (222 subsets); (4) four weeks of calibration followed by eight weeks of deployment (222 subsets); (5) two weeks of calibration followed by 16 weeks of deployment and another two weeks of calibration (166 subsets); (6) two weeks of calibration followed by 24 weeks of deployment and another two weeks of calibration (110 subsets). These calibration scenarios are evaluated both on 2 min averaged data and on daily averages for comparison with the performances standards of the certifications protocols. For example, the first scenario described above uses the first week and the last week of n a 10 weeks period to calibrate the sensors against the Fidas 200S, and evaluates the performances of this calibration on the eight remaining weeks. There are 236 possible subsets of 10 consecutive weeks in the dataset starting on different day for each of these subset, the RMSE of the evaluation period is calculated and used to plot the Box and Whisker plots used throughout this paper and described below.

### 2.4 Box and Whisker plot

Given the amount of data present in each of the analyses, Box and Whiskers plots were used throughout to clearly illustrate the range of the data collected. The line inside the boxes represents the median value, the box boundaries represent the 25th and 75th percentiles, the whiskers extend to 1.5 times the interquartile range and the data points outside of this range are considered as outliers, drawn as dots.

### 2.5 Statistical analysis

The data from the Fidas 200S and the PM sensors were averaged every 10 s for the incense experiment and for the initial analysis. It was then averaged every 2 min using the arithmetic mean. Hourly and daily averages were also used for comparison purposes. The data used are openly available at https://doi.org/10.5281/zenodo.7198378 and the R code used for the data analysis is openly available at https://doi.org/10.5281/zenodo.7261417. Faulty sensors were detected through the data process described in Bulot *et al.* [5]. Two Plantower PMS5003 reported high peaks that were not reported by any other sensors (including the other Plantower PMS5003 duplicated present in the same air quality monitor). These two sensors were excluded from further analysis. One Sensirion SPS30 reported values with a mean of zero verified by a *t*-test (t = 233.3 and p < 2.2e - 16) and this was discarded from further analysis.

The metrics used to evaluate the sensors are: the expanded uncertainty, the between unit uncertainty and the RMSE.

The guide to the demonstration of equivalence of ambient air monitoring methods (36) defines the expanded uncertainty as the combined relative uncertainty of the sensor studied multiplied by a coverage factor of 2 for a 95% confidence interval, expressed in %. The combined relative uncertainty *w*_*c*_ is defined as:

(1) $$w_{c}^{2}\left(y_{i}\right)=\frac{u_{CR}^{2}\left(y_{i}\right)}{y_{i}^{2}}$$

where *y*_*i*_ is the concentration at the limit value (25 μg/m^3^ for MCERTS for Indicative Ambient Particulate Monitors [4]) and *u*_*CR*_ is the combined uncertainty, calculated as the sum of the bias and the random error and defined at a given concentration *x*_*i*_ by:

(2) $$u_{CR}^{2}\left(x_{i}\right)=\frac{RSS}{n-2}-u^{2}\left(x_{i}\right)+{\left[a+\left(b-1\right)x_{i}\right]}^{2}$$

where *RSS* is the sum of residuals, *n* is the number of data points included, *a* and *b* are respectively the intercept and the slope of the linear model between the sensor and the reference method, determined through an orthogonal least square regressions [37] and *u*^*2*^*(x*_*i*_*)* is the uncertainty of the reference method. The uncertainty of the reference method can be taken as:

(3) $$u^{2}\left(x_{i}\right)=\frac{u_{bs,RM}}{\sqrt{2}}$$

where *u*_*bs,RM*_ is the between unit uncertainty of the reference instrument expressed in μg/m^3^. For the Fidas 200S, *u*_*bs,RM*_ = 0.48 μg/m^3^ [38].

The slopes are calculated for the orthogonal least squares, following the recommendation of the Guide for the Demonstration of Equivalence of Ambient Air Monitoring Methods [36]. In this case, the slope b is given by:

(4) $$b=\frac{S_{yy}-S_{xx}+\sqrt{{\left(S_{yy}-S_{xx}\right)}^{2}+4{\left(S_{xy}\right)}^{2}}}{2S_{xy}}$$

with

(5) $$S_{xx}=\overset{n}{\underset{i=1}{\sum }}{\left(x_{i}-\bar{x}\right)}^{2},S_{yy}=\overset{n}{\underset{i=1}{\sum }}{\left(y_{i}-\bar{y}\right)}^{2},S_{xy}=\overset{n}{\underset{i=1}{\sum }}\left(x_{i}-\bar{x}\right)\left(y_{i}-\bar{y}\right)$$

where *x*_*i*_ and *y*_*i*_ are the measurements at the instant *i* and $\bar{x}$ and $\bar{y}$ are the mean values of *x* and *y* over the measurement series.

An expanded uncertainty of 25% is required for MCERTS for UK PM [2], which is the certification required for reference instruments used for example as part of the Automatic Urban and Rural Network (AURN) network, and for MCERTS for Continuous Ambient Air Monitoring System [39] and of 50% for MCERTS for Indicative Ambient Particulate Monitors [4] as defined by the EU Data Quality Objectives [3]. In this study, the expanded uncertainty is mostly used to characterise daily averages to compare them to the Data Quality Objectives. It is also used to compare daily, hourly and 2 min averages. It is important to note that the expanded uncertainty is generally only used to compare daily measurements to verify compliance to legal limits (which are set as daily or yearly averages).

The between unit uncertainty is defined by:

(6) $$u_{bu}^{2}=\frac{\sum_{i=1}^{n}{\left(y_{i,1}-y_{i,2}\right)}^{2}}{2n}$$

where *y*_*i,1*_ and *y*_*i,2*_ are the measurements taken by two units and n is the number of data point pairs for the two units considered. It is used as a measure of the accuracy of the sensors. A between unit uncertainty of 5 μg/m^3^ is required for MCERTS for Indicative Ambient Particulate Monitors [4] and of 2.5 μg/m^3^ for MCERTS for Continuous Ambient Air Monitoring System [39] and for MCERTS for UK PM (*2*). The between unit uncertainty is used here to compare different time averages and to evaluate the 2 min data.

The RMSE is mostly used in this study to characterise the different calibration models at 2 min resolution and for two measurements X and Y is calculated as $RMSE=\sqrt{mean{(\left(X-Y\right)}^{2})}$.

## 3 Results

The first part of this section presents the characterisation of the behaviour of low-cost PM sensors at 10 s resolution and based on these results the determination of a suitable time averaging period for further analyses is determined. The existing certifications for PM instruments utilise daily data, and most reference instruments produce hourly data. Therefore, a comparison of the performances of the sensors at these different temporal resolutions was presented for uncalibrated data. Through the second part, different calibration methods are characterised, in three steps: (1) the sensors are calibrated on the data from the first 14 days of the deployment and evaluated on the following 40 days, presented in Supplementary information Section 3.2; (2) the evolution of the performances of the calibration methods is evaluated on a month-to-month basis; (3) to characterise robustly the calibration methods, they are then evaluated on every subset available in the dataset of 14 days followed by 40 days of validation, defining a cross-validation of the calibration models developed. This robust characterisation enabled selection of methods with which, in the third part of this section, the six calibration scenarios described in the methods were characterised and compared to reflect how sensors could be calibrated in real-life. These scenarios were first characterised on the 2 min data and finally on daily data to enable comparison of the results obtained with the requirements of the MCERTS protocols.

### 3.1 Time averaging

#### 3.1.1 Transient peaks of PM

During the early stages of the analysis, a delay between the peaks recorded by the Fidas 200S and the sensors at 10 s resolution was noticed. To characterise this delay, incense sticks were simultaneously lit close to the inlet of the Fidas 200S and to the inlet of the sensors, for 30 s every hour between 10.0 and 15.00. The time series of the PM_2.5_ concentrations during this experiment are presented in Fig. 1. The concentrations were reported every 10 s and scaled for comparison. Five peaks were generated and measured by both the low-cost sensors and the Fidas 200S. The upper panel gives an overview of the experiment conducted while the lower panel gives a magnified view on one of the peaks generated. When focusing on the individual peaks resulting from a single transient incense exposure, each consists of two individual events. The first peak is smaller and corresponds to the moment the incense was lit before being moved close to the inlets. The second peak corresponds to the longer burn of the incense. These time series clearly illustrate a delay between the second peak reported by the Fidas 200S and the peaks reported by the sensors. This delay was characterised for each sensor, the results are presented in Supplementary Figure S8. The delay was variable with time (between 0 s and 50 s), seemingly random across peaks, and most likely linked to external environmental factors. Several factors conceivably caused this delay but the distance between the inlet of the sensors and that of the Fidas 200S, although small, could be significant when considering timescales as short as 10 s. Because of the variable nature of the delay, it was not possible to simply correct for it by shifting the data for a certain amount of time, and instead, methods such as Derivative Dynamic Time Warping (DDTW) would be recommended to use data at these time scales as used in Bulot *et al.* [6]*.*

Maierhofer *et al.* [40] detailed the theoretical variability of PM concentrations at small scales: counting statistics of small number of particles cannot accurately represent the particles size distribution and other minor errors may arise from the distribution of the particle density. To obtain a 5% theoretical uncertainty for PM_2.5_, it is necessary to sample a minimum mass of 400 pg. This means that assuming a flow rate of 0.5 L/min (which is the flow rate of the Alphasense OPC-R1, while other sensors studied in this study have a lower flow rate, not specified by their manufacturer), it will take a sampling time of circa 24 s to measure PM concentrations in the order of 2 μg/m^3^. For comparison, the Fidas 200S has a flow rate of 4.8 L/min and would therefore require about 2.5 s of sampling to collect the 400 pg required for a 5% theoretical uncertainty at this concentration level. Maierhofer *et al.* [40] used for their calculations a log-normal monodisperse distribution of particles with a geometric mean of 0.3 μm and a geometric standard deviation of 1.7 μm for the particle distribution, while during the period of this study, the geometric mean is of 0.53 μm and a geometric standard deviation of 1.46 μm (calculated from the particle size distribution measured by the Fidas 200S and presenting little variation throughout the study), which influences the theoretical uncertainty. To avoid complications from the theoretical uncertainty described here and because of the delay observed with the transient peaks of incense, it was decided to use data averaged over 2 min (2 min mean) for the rest of this study.

#### 3.1.2 Impact of the time averaging on the certification metrics

In this section, the metrics were calculated on the sensors data, for all the possible combinations of 40 successive days in the period of study using (1) 2 min mean; (2) 1 h mean; (3) 24 h mean, without calibration as the Sensirion SPS30 obtained the MCERTS for Indicative Ambient Particulate Monitors. The result of this section focused on the expanded uncertainty and the between unit uncertainty as these are the two metrics required by the MCERTS for Indicative Ambient Particulate Monitors (4), the Root Mean Square Error (RMSE) is also given for comparison.

**Fig. 3:**
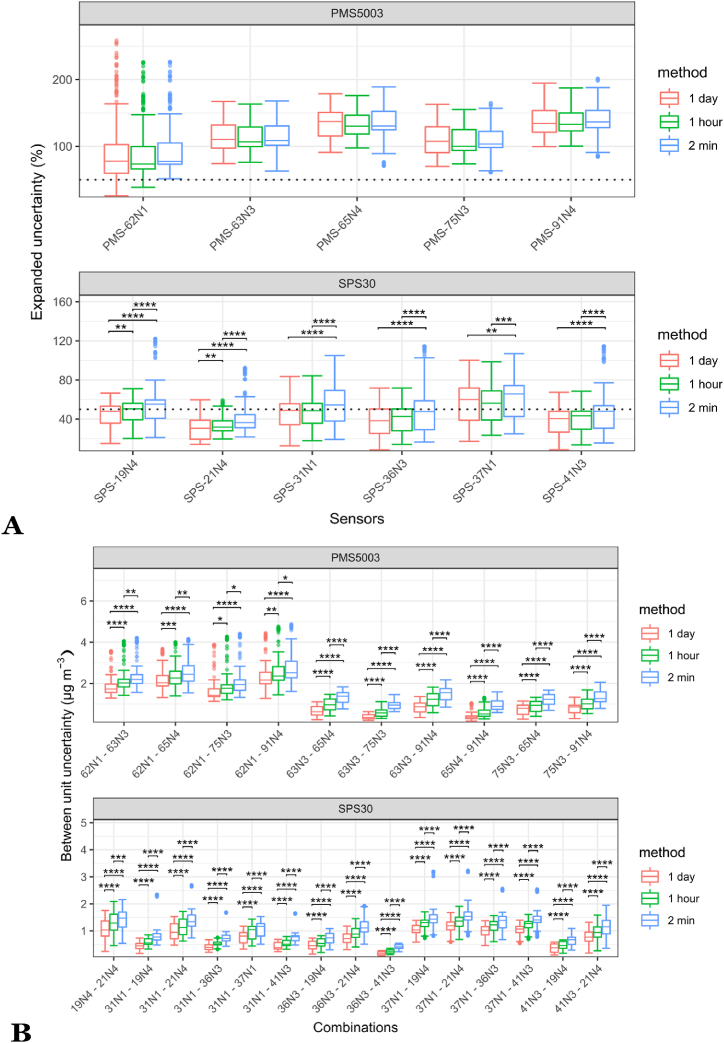
(A) Expanded uncertainty and (B) between unit uncertainty (of all the possible combinations of sensors of the same model) obtained by the different Sensirion SPS30 (SPS- xxNx) and Plantower PMS5003 (PMS-xxNx) during each possibility of 40 consecutive days during the study (n = 266 40 days periods in total, represented as Box and Whisker plots), for daily, hourly and 2 min data. In (b), the letters PMS and SPS were deleted to not overload the labels. The dotted line represents the 50% expanded uncertainty threshold required for MCERTS for Indicative Ambient Particulate Monitors. Significant differences are reported: *p < 0.1, **p < 0.01, ***p < 0.001, ****p < 0.0001. Groups have been tested for normality with a Shapiro test, for equal variance with a Levene test and have been compared accordingly using a *t*-test.

Fig. 3 shows the results obtained by each of the sensors for different time-averaging intervals (2 min, 1 h and 24 h means). Overall, for the expanded uncertainty for 2 min data, the Sensirion SPS30 obtained a 25th quantile, median and 75th quantile of 34, 50 and 64% respectively, and the Plantower PMS5003 of 99, 125 and 138%, to compare to the 50% requirement from the MCERTS for Indicative Ambient Particulate Monitors. For the between unit uncertainty for 2 min data, on Fig. 3B, which shows the between-unit uncertainty of each possible pair of a given model of sensor, the Sensirion SPS30 obtained a 25th quantile, median, and 75th quantile of 0*.*2, 1*.*1 and 1*.*6 μg/m^3^ respectively, and the Plantower PMS5003 of 0*.*5, 1*.*2 and 2*.*0 μg/m^3^, compared to 2*.*5 μg/m^3^ for the MCERTS for Indicative Ambient Particulate Monitors. Fig. 3B shows that there are clear statistical differences between the different time averaging periods while this is not the case for the expanded uncertainty.

For the Plantower PMS5003, there are no statistically significant differences between the results obtained by the three different averaging periods. For the Sensirion SPS30, there are significant differences between the different averaging periods for all individual sensor units apart for SPS-38N2. For this sensor model, the expanded uncertainty is only marginally lower for the daily average, and the hourly average than for the 2 min average.

For the Plantower PMS5003, the expanded uncertainty was consistently greater than 50% and above 100% for most sensors while for the Sensirion SPS30, at 2 min averages, the expanded uncertainty was mostly below 50% for three sensors out of seven (SPS–21N4, SPS- 36N3, and SPS-41N3), it was around 50% for two other sensors (SPS–19N4, SPS-31N1), and it was greater than 50% for the two remaining sensors. For daily averages, three Sensirion SPS30 were consistently below the 50% threshold. For the between unit uncertainty, it was below the threshold of 5 μg/m^3^ for all the sensors across both manufacturers. Daily averages obtain a better score for this metric with a reduced interquartile range compared to hourly averages, and hourly averages obtained a better score than the 2 min data.

**Fig. 4:**
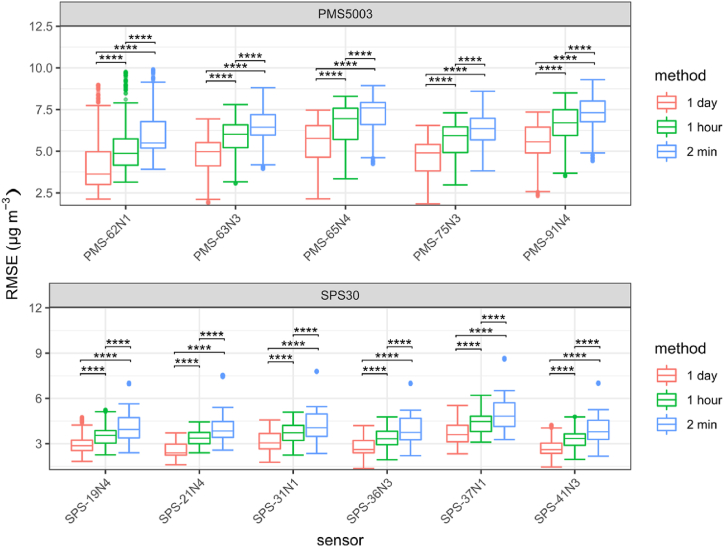
Root Mean Square Error (RMSE) obtained by the different Sensirion SPS30 (SPS-xxNx) and Plantower PMS5003 (PMS-xxNx) during each possibility of 40 consecutive days during the study (n = 266 40 days periods in total, represented as Box and Whisker plots), for daily data, hourly data and data averaged every 2min. Significant differences are reported: *p < 0.1, **p < 0.01, ***p < 0.001, ****p < 0.0001. Groups have been tested for normality with a Shapiro test, for equal variance with a Levene test and have been compared accordingly using a *t*-test.

Fig. 4 presents the same results for the RMSE. Overall, for the RMSE for 2 min data, the Sensirion SPS30 obtained a 25th quantile, median and 75th quantile of 5*.*7, 6*.*7 and 7*.*6 μg/m^3^ respectively and the Plantower PMS5003 of 3*.*4, 4*.*0 and 4*.*8 μg/m^3^. For the Plantower PMS5003 the RMSE was lower with daily averages than with the other averaging period for most sensors with similar or narrower interquartile ranges. For the Sensirion SPS30 the situation was more mixed with the metrics for the daily averages being lower for all sensors but with the interquartile range increasing compared to the 2 min data. The RMSE obtained by the Sensirion SPS30 sensors are lower than those obtained by the Plantower PMS5003.

### 3.2 Calibration methods selection

#### 3.2.1 Evolution of the performance of the calibration through time

**Fig. 5:**
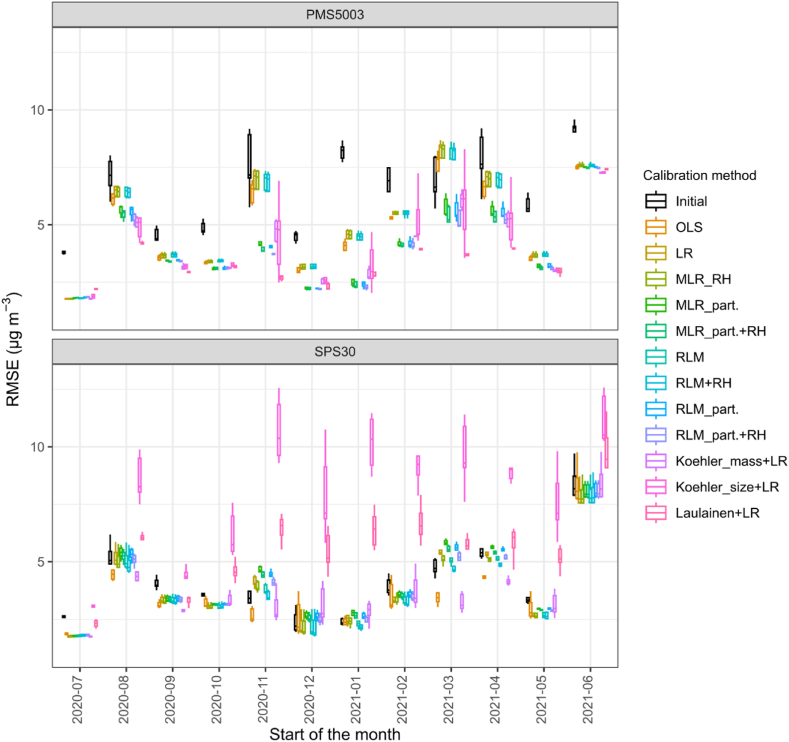
Box and Whisker plot of the monthly evolution of the Root Mean Squared Error (RMSE) obtained for the different calibration methods considered for every sensor of the same model. The calibration methods were trained on the first two weeks of the dataset. The Box and Whisker boxes represent the values obtained by the different sensors of each model per month. OLS: Orthogonal Least Square; LR: ordinary least square; MLR_RH: Multi-Linear Regression with Relative Humidity (RH); MLR_part.: Multi-Linear Regression on particle number concentration; MLR_part.+RH: Multi-Linear Regression on particle number concentration and RH; RLM: Robust Linear Model.

Fig. 5 presents the monthly evolution of the RMSE of the calibration methods trained on the first two weeks of the dataset when applied to the rest of the dataset. The performances varied through the year. The calibration methods generally obtained a higher RMSE during the months of August, March and June than for the other months of the study. For the Plantower PMS5003, any of the calibration methods resulted in an improvement of the performances of the sensors compared to the initial (i.e. non-calibrated) situation for most months, although not as good as in July 2020. For March 2021, some methods increased the RMSE. The methods based on particle number concentrations and on physical correction of relative humidity (RH) obtained better scores than the other regression methods used. Conversely, for the Sensirion SPS30, whose initial RMSE were lower, month after month, than the Plantower PMS5003, the calibration methods did not necessarily improve the performances compared to the initial situation. Koehler size + LR increased the RMSE for all months and Laulainen + LR increased the RMSE for most months compared to the initial situation. For the Sensirion SPS30, LR decreased the RMSE compared to the initial situation, although as low as in July 2020 when the calibration was conducted.

#### 3.2.2 Robust method selection

**Fig. 6:**
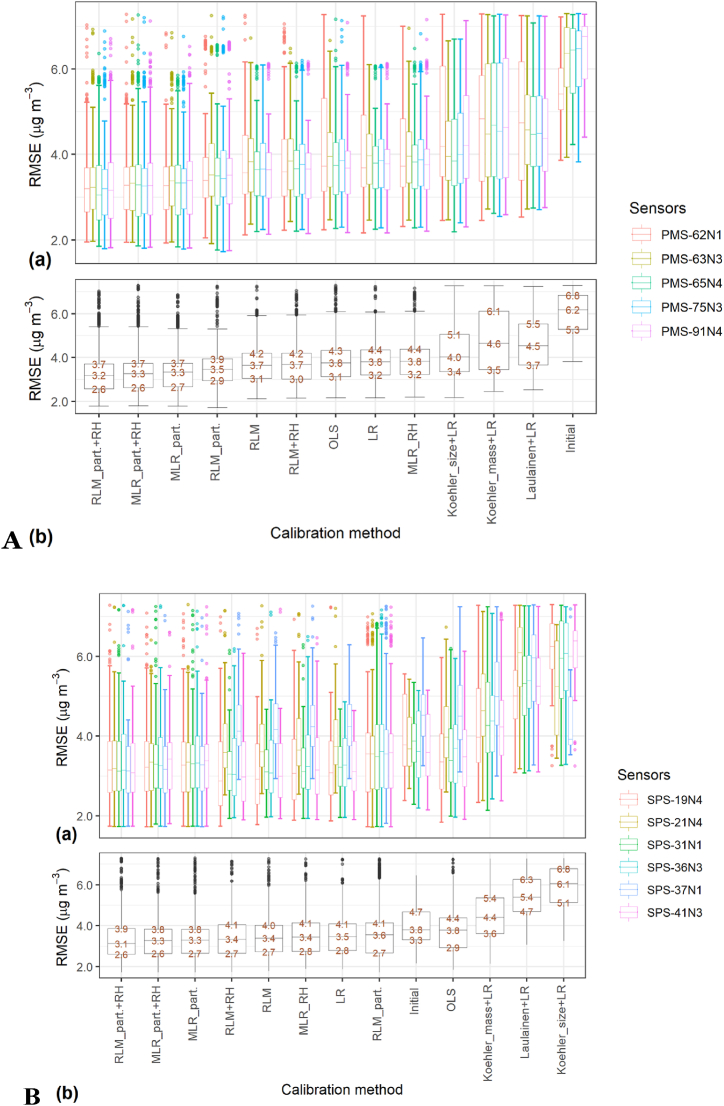
Box and Whisker plot of the Root Mean Squared Error (RMSE) obtained for the different calibration methods considered for each (A) Plantower PMS5003; (B) Sensirion SPS30; individually for all the combinations of two weeks of calibration followed by 40 days of evaluation during the study (n = 252). The upper panels (a) represent the RMSE obtained by individual sensors and the lower panels (b) represent the overall RMSE per sensor model. The Box and Whisker boxes represent the values obtained by each individual sensors per month and per calibration method. The values on the lower panel, from bottom to top, are the 1st quartile, the median and the 3rd quartile. Only data between the 10th and the 90th centile of the data are displayed.

To further this preliminary analysis, the calibration methods were evaluated on all possible combinations within the dataset of two weeks of calibration with 40 days of evaluation. This enabled to robustly identify the calibration methods that would be most appropriate. The results are presented in Fig. 6A for the Plantower PMS5003 and summarised in Supplementary Table S4 and in Fig. 6B and summarised in Supplementary Table S5 for the Sensirion SPS30.

For the Sensirion SPS30, the correction methods based on Koehler and Laulainen corrections with Linear Regression based on ordinary least square (LR) or Orthogonal Least Square (OLS) led to an increase of RMSE compared to the initial situation, without calibration. The method that obtained the best median RMSE and interquartile range was the Robust Linear Model (RLM) on particle numbers with RH, closely followed by Multi-Linear Regression (MLR) on particle numbers with RH and without RH. For the MLR models, the inclusion of RH did not improve the results, while for the RLM models, the inclusion of RH improved the results obtained. The calibration methods based on particle number concentration reduced the differences of performances between individual sensors that were present prior to calibration (initial situation) while the calibration methods on PM mass concentration preserved these differences. Nevertheless, for the calibration methods that improved the RMSE compared to the initial situation, there were only small differences between their respective median RMSE.

For the Plantower PMS5003, all the calibration methods tested improved the RMSE compared to the initial situation. The calibration methods based on Koehler and Laulainen corrections performed the worst compared to the other tested methods and with higher median RMSE and wider interquartile ranges. Similar to the Sensirion SPS30, the three methods that performed the best were Robust Linear Model (RLM) on particle numbers with RH and Multi-Linear Regression (MLR) on particle numbers with and without RH, as for the Sensirion SPS30. Conversely to the Sensirion SPS30, the calibration methods used on the Plantower PMS5003 substantially improved the RMSE compared to the initial situation. It is important to note that the median RMSE was 3*.*8 μg/m^3^ for the Sensirion SPS30 and 6*.*8 μg/m^3^ for the Plantower PMS5003, while the best median RMSE obtained after calibration was 3*.*0 μg/m^3^ for the Sensirion SPS30 and 3*.*1 μg/m^3^ for the Plantower PMS5003.

Overall, for both sensor models, the methods using the particle number concentrations as calibration variables obtained better results than the methods focusing on the PM_2.5_ mass concentrations.

### 3.3 Calibration scenarios

#### 3.3.1 Calibration scenarios and 2 min data

**Fig. 7:**
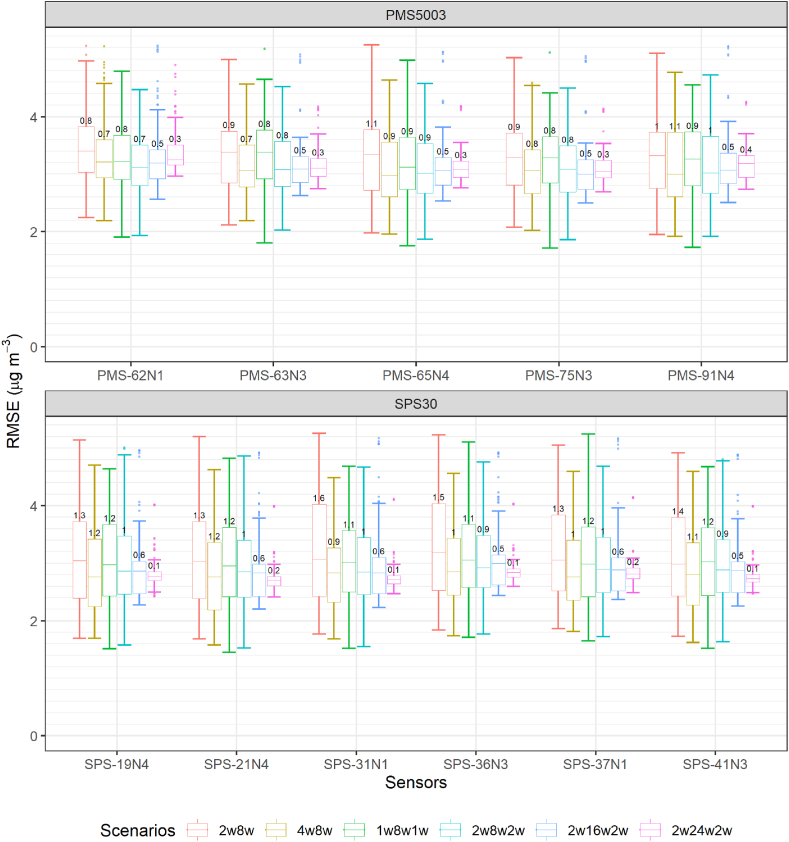
Box and Whisker plot of the Root Mean Squared Error (RMSE) obtained for the Robust Linear Model on particle number concentrations (RLM part.+RH) with Relative Humidity (RH) for every sensor of the same model for different scenarios of calibration. The calibration method was trained and evaluated on the 2 min data. 2w8w: 2 weeks of calibration followed by 8 weeks of evaluation; 4w8w: 4 weeks of calibration followed by 8 weeks of evaluation; 1w8w1w: 8 weeks of evaluation with 1 week of calibration before the evaluation and 1 week after; 2w8w2w: 8 weeks of evaluation with 2 weeks of calibration before the evaluation and 2 weeks after; 2w16w2w: 16 weeks of evaluation with 2 weeks of calibration before the evaluation and 2 weeks after; 2w24w2w: 24 weeks of evaluation with 2 weeks of calibration before the evaluation and 2 weeks after (2w8w: n = 236; 4w8w: n = 222; 1w8w1w: n = 236; 2w8w2w: n = 222; 2w16w2w: n = 166; 2w24w2w: n = 110). The values above the Box and Whisker boxes are the interquartile range of the box. The Box and Whisker boxes represent the values obtained by the different sensors of each model during all the combinations possible of the scenarios during the study.

In this subsection, six calibration scenarios were assessed using all the possible subsets of data. The six scenarios considered are: (1) two weeks of calibration followed by eight weeks of deployment (2w8w - 236 subsets); (2) four weeks of calibration followed by eight weeks of deployment (4w8w - 222 subsets); (3) one week of calibration followed by eight weeks of deployment and another week of calibration (1w8w1w - 236 subsets); (4) two weeks of calibration followed by eight weeks of deployment and another two weeks of calibration (2w8w2w - 222 subsets); (5) two weeks of calibration followed by 16 weeks of deployment and another two weeks of calibration (2w16w2w - 166 subsets); (6) two weeks of calibration followed by 24 weeks of deployment and another two weeks of calibration (2w24w2w - 110 subsets). For clarity and based on our earlier results (Fig. 6), only the RLM on particle number concentrations with RH is used in this section. Fig. 7 presents the results of this evaluation. As perhaps expected, 4w8w obtained better RMSE than 2w8w and 2w8w2w obtained better median RMSE than 1w8w1w due to the increased calibration duration.

For both sensor models, excluding the 2w8w and 1w8w1w scenarios, there was little differences between the median RMSE obtained by the four other calibration scenarios (*<*0*.*2 μg/m^3^) with scenario 4w8w generally obtaining better results than the other scenarios for the Plantower PMS5003 and scenario 2w24w2w for the Sensirion SPS30. However, the scenarios that consistently obtained the lowest interquartile ranges were 2w16w2w and 2w24w2w, with substantial differences compared to the other scenarios interquartile ranges. Notably, for the Sensirion SPS30, scenario 2w24w2w obtained an interquartile range *<*0*.*2 μg/m^3^ demonstrating an excellent reliability. As a comparison, the interquartile range obtained by the Sensirion SPS30 through the robust method selection for this calibration method was 1*.*3 μg/m^3^.

#### 3.3.2 Calibration scenarios and daily performances

**Fig. 8:**
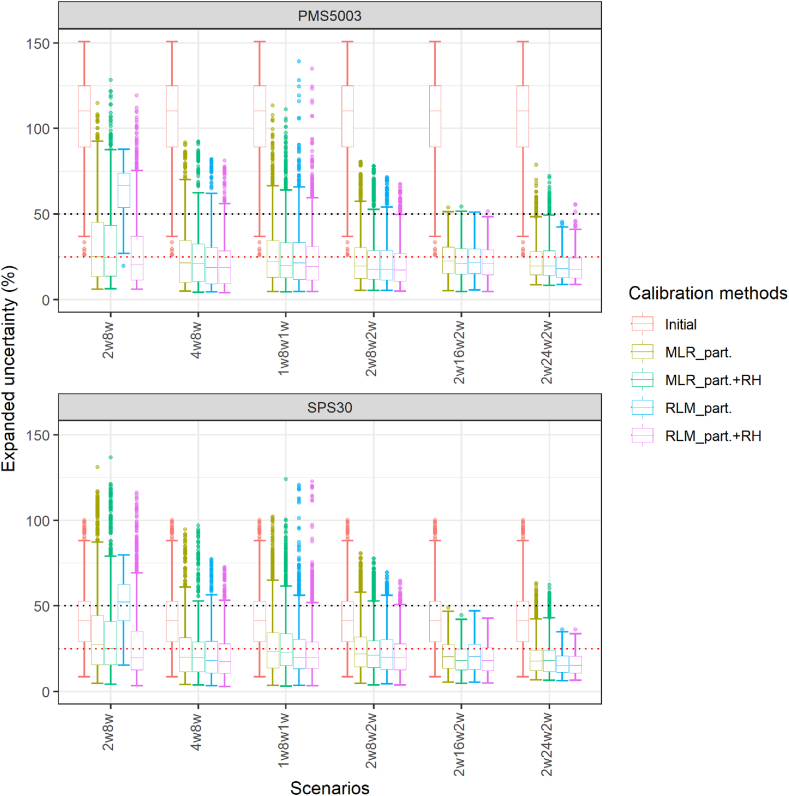
Box and Whisker plot of the expanded uncertainty on daily averages, obtained for the calibration methods considered for the two sensor models for different scenarios of calibration. The calibration methods were trained and applied and the 2 min data and then averaged daily. 2w8w: 2 weeks of calibration followed by 8 weeks of evaluation; 4w8w: 4 weeks of calibration followed by 8 weeks of evaluation; 1w8w1w: 8 weeks of evaluation with 1 week of calibration before the evaluation and 1 week after; 2w8w2w: 8 weeks of evaluation with 2 weeks of calibration before the evaluation and 2 weeks after; 2w16w2w: 16 weeks of evaluation with 2 weeks of calibration before the evaluation and 2 weeks after; 2w24w2w: 24 weeks of evaluation with 2 weeks of calibration before the evaluation and 2 weeks after (2w8w: n = 236; 4w8w: n = 222; 1w8w1w: n = 236; 2w8w2w: n = 222; 2w16w2w: n = 166; 2w24w2w: n = 110). The Box and Whisker plot represents the values obtained by all the possible subsets of each scenario during the study for each sensor model for the different calibration methods considered. The black dotted line corresponds to an expanded uncertainty of 50% and the red dotted line to an expanded uncertainty of 25%.

The calibration methods were applied to the 2 min data as before, for each of the six calibration scenarios, and after that, the calibrated data from the sensors were averaged per day to compute the expanded uncertainty and compare the values obtained to the Data Quality Objectives of the MCERTS for UK PM (expanded uncertainty of 25%) and the MCERTS for Indicative Ambient Particulate Monitors (expanded uncertainty of 50%). Fig. 8 presents the results for the Plantower PMS5003 and the Sensirion SPS30, respectively. For the Plantower PMS5003, scenarios 2w8w2w, 2w16w2w and 2w24w2w enable the calibration methods based on particle number concentrations to stay below the 50% threshold for expanded uncertainty and mostly below the 25% threshold. The RLM on particle number concentration and RH obtains the best results with most of the sensors below or around 25% expanded uncertainty. Scenario 1w8w1w obtained similar results but with more outliers than 2w8w2w. The 2w8w and 4w8w scenarios, while performing less well than the two other scenarios, still enabled significant improvements compared to the initial situation, uncalibrated, and the calibration methods using particle numbers had their interquartile range below 50%. The results were similar for the Sensirion SPS30.

**Fig. 9:**
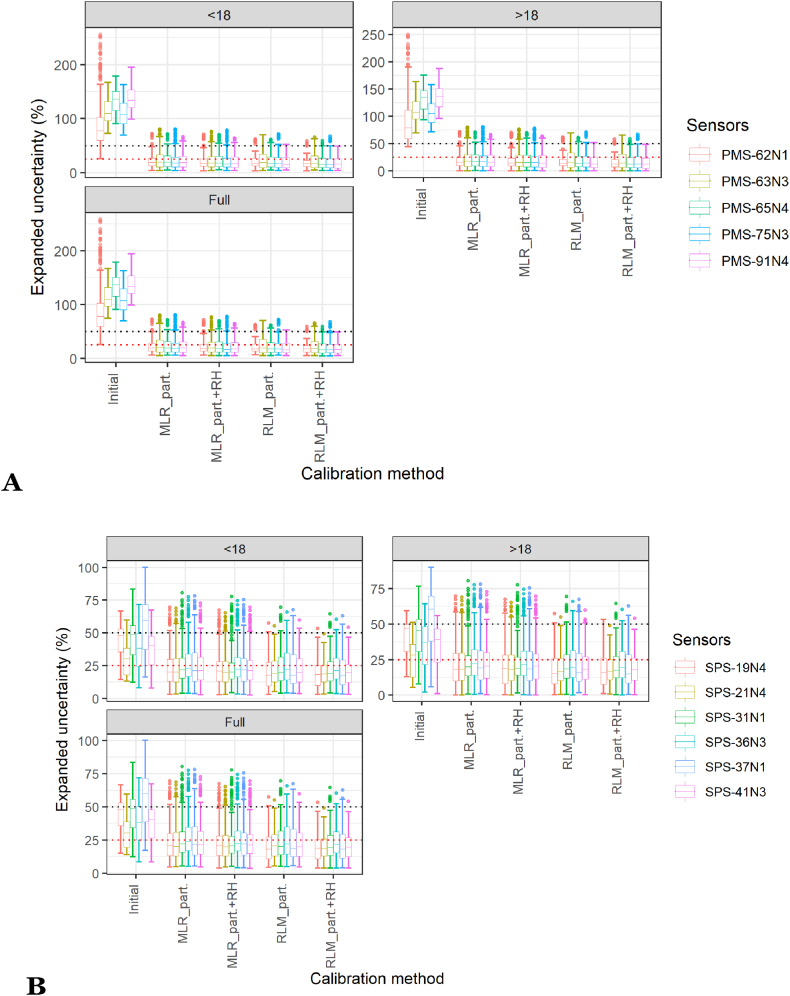
Box and Whisker plot of the expanded uncertainty on daily averages, obtained for the calibration methods considered for every sensor of the: (A) Plantower PMS5003; (B) Sensirion SPS30 model for scenario 2w8w2w for the three types of datasets required by the MCERTS for Indicative Ambient Particulate Monitors: the full dataset and the two subsets of data obtained by splitting the full dataset by concentrations greater than or lesser than 18 μg/m^3^. The calibration methods were trained and applied to the 2 min data and then averaged daily. 2w8w2w: 8 weeks of evaluation with 2 weeks of calibration before the evaluation and 2 weeks after; (n = 55). The Box and Whisker plot represents the values obtained by all the possible subsets of each scenario during the study for each sensor. The black dotted line corresponds to an expanded uncertainty of 50% and the red dotted line to an expanded uncertainty of 25%.

The certification for Indicative Ambient Particulate Monitors requires that the Data Quality Objectives are achieved for the full dataset and for two subsets of the data split by concentration greater than and less than 18 μg/m^3^. Fig. 9 presents the expanded uncertainty obtained for the different calibration methods on scenario 2w8w2w for the Plantower PMS5003 and the Sensirion SPS30 for the three types of datasets required by the certification. The sensors exhibited similar results over the three types of datasets and all the sensors passed the Data Quality threshold of 25% almost consistently. Supplementary Table S6 details the results obtained for the expanded uncertainty for the calibration scenario 2w8w2w for MLR and RLM on particle number and with and without RH. All the calibration methods improved substantially the expanded uncertainty to levels matching the Data Quality Objectives for indicative measurements for the two sensor models.

## 4 Discussion

**Fig. 10:**
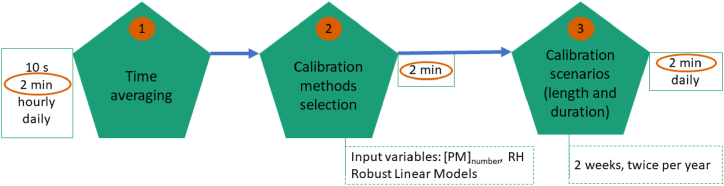
Summary of the analysis conducted and main results.

Fig. 10 summarises the different analysis conducted in this paper and the main results obtained. In this study, the behaviour of two low-cost PM sensors, Plantower PMS5003 and Sensirion SPS30, at 10 s average was studied initially and the inherent uncertainty linked to the mass of PM sampled at low concentrations led to the recommendation to use a 2 min averaging window. Then, a comparison between the 2 min and hourly and daily data was presented focusing on the differences in expanded uncertainty, between unit uncertainty and RMSE. Different calibration methods were robustly tested on different subsets of the dataset and the evolution of the performances of these methods monthly was analyzed. Six calibration scenarios were then tested to further improve the calibration methods proposed. Finally, the calibration scenarios were used to evaluate the performances of the sensors in comparison with the performance requirements of reference instruments. The performances of calibration methods and calibration scenarios have been validated, enabling low-cost PM sensors to obtain reference-grade performances at a 2 min resolution, with calibration required at most every six months.

Our findings concerning the minimum time averages and the delay experienced by the sensors have implications for the potential use of these sensors. Following Maierhofer *et al.* [40], the theoretical precision of the PM sensors depends on the total mass of PM they sampled and, therefore will be driven by the time averaging and the background level of PM concentrations to be measured. Additionally, in 2019, Bulot *et al.* [5] demonstrated that, in Southampton, the sensors were less reliable at concentration levels ≈ *<*10 μg/m^3^.

For qualitative studies, it may be possible to use a temporal resolution *<*2 min but for quantitative studies, a temporal resolution of 2 min or more is required especially when the variations of PM observed are in the order of 1 μg/m^3^ or less as observed here. Given the recent update of the World Health Organisation (WHO) guidelines for PM_2.5_ with an annual concentration of 5 μg/m^3^ and 15 μg/m^3^ as a 24 h mean, these low concentrations will likely be encountered more often in the future. Additionally, if these sensors are to be used to check compliance with the WHO guidelines, they will need to be able to measure reliably at low concentrations. The problem did not arise in Bulot *et al.* [6], where the same air quality monitors were tested at a 10 s resolution in the laboratory in an well-mixed environmental enclosure with peaks of incense and candle PM generated smoke with concentrations 10–100 μg/m^3^ so the required sampling time for a theoretical uncertainty of 5% was between 0*.*4 to 4 s. Alternately, the limitations described to measure at a temporal resolution below 2 min linked to the total number of particles measured and hence to the sensing volume of the sensors and their airflow means that it is unlikely that smaller sensors will appear, and the current size of the sensors will be a hard limit to the improvement of their performances. Additionally, if the levels of pollution decrease to levels complying with the WHO guideline of 5 μg/m^3^, sensor precision will be impacted, and this is also true for reference-grade instruments.

Here, the PM_2.5_ concentrations observed ranged between 0.4 μg/m^3^ and 70.2 μg/m^3^ with a median value of 5.5 μg/m^3^, which is relatively low compared to other field studies. Badura *et al.* [41] showed that four models of sensors, including a Plantower PMS7003, obtained higher relative error and larger dispersion for PM_2.5_ concentrations <20–30 μg/m^3^. Johnson *et al.* [42] demonstrated that different models of Shinyei PM sensors were not reliable at concentrations below 40 μg/m^3^ while the obtained good performances above. However, it is to be noted that compared to the sensors we tested here, the Shinyei relies on a heated resistor to draw the particles in while the sensors have a built-in fan. These further highlight the usefulness of the method proposed here to obtain performance grade performances even at low levels of pollution. For high concentrations, Zheng *et al.* [43] tested Plantower PMS3003 in high and low concentrations outdoor environment, one in the US and one in India and observed that, for concentrations > ∼125 μg/m^3^, the sensors presented a non-linear behaviour that could be captured by a quadratic fit. The clipping observed on the data corrected for some machine learning techniques, in Supplementary Figure S7, shows that the range of PM concentrations experienced during the calibration campaign can impact the performances of calibration methods. The range of concentrations experienced during the calibration period should be representative of the deployment conditions, especially for advanced correction methods.

Other seasonal parameters will also impact the performances, for instance, if the calibration happens during the heating season and then the sensors are used during the non-heating season, the sources, composition and range of PM will vary. Hence, when designing a measurement campaign, it is important to define the type of PM pollution of interest (e.g. impact of the heating season) in order to define the best periods for calibrating the sensors (before and after the deployment). Nonetheless, having several calibration periods spaced throughout the year may help improve the performances of the sensors. It is interesting to note that the calibration scenarios on longer periods of time (16 or 24 weeks - 4 or 6 months) obtained narrower distributions than the shorter ones and generally with lower means. This may be because the duration of the deployment (16 or 24 weeks - 4 or 6 months) enables the two calibrations to be further apart timewise. This have for effect to provide the sensor with a more varied calibration data and to incorporate some of the seasonal variations of PM characteristics which is relevant given the sensitivity of the sensors to PM composition. A similar idea has been explored in Lee *et al.* [26].

Only a few studies have examined the performances of low-cost PM sensors at the temporal resolution examined here. Mahajan *et al.* [18] studied nine duplicates of Plantower PMS5003 against a GRIMM EDM 107 with the sensors sampling every 30 s, however the time aver-aging period used for the analysis is not specified, the study only lasted for 15 h, the results obtained for the linear regression model obtained very low slopes, around 0.03, which had not been observed in other studies and the data are not available for verification. Their study recommended SVM, but they only evaluated the method by splitting their available dataset into a training and a test dataset hence it cannot infer whether SVM would work well on another dataset. Di Antonio *et al.* [27] studied the calibration of an Alphasense OPC-N2 at 60 s average over six weeks in an outdoor collocation against a Fidas 200S and proposed a correction method based on the correction of the hygroscopic growth of particles using the Koehler theory. The Alphasense OPC-N2, is a single particle counter with capacity to differentiate particles into 16 different size bins while in comparison, there is concern in the literature that the Plantower PMS5003 and the Sensirion SPS30 are not capable of accurately differentiating particles sizes or only capable of differentiating a relatively small number of different bin sizes [7,31,44]. This is further supported in the case of the Sensirion SPS30 by the results obtained in the present study, given the correlations observed between the different bin size of this sensor. As such, it is not clear whether the correction/calibration method applied to the Alphasense OPC-N2 sensor can be applied to other sensor models without the size resolution for particle number concentrations. Crilley *et al.* [45] also developed a correction method for the Alphasense OPC-N2 at 5 min resolution based on the hygroscopic growth of particles applied directly to the mass of particles rather than on the particle number concentration. The negative results obtained in this study for the Koehler correction on mass further support the hypothesis that the correction methods developed for single Optical Particle Counter (OPC) may not be suitable for other low-cost PM sensors, although parallel studies with an OPC would be needed to confirm this. Despite the limitations noted on the particle number concentrations reported by the sensors, the calibration methods that obtained the best results, in all cases for the Plantower PMS5003 and to a lesser extent for the Sensirion SPS30 at 2 min mean, were the methods involving particle number concentrations with and without RH. This shows that it is preferable to use these measurements instead of the software-calculated mass concentrations to limit the influence of potential built-in algorithms of the sensors. Similar to this current study, Cavaliere *et al.* [20] examined the calibration of a Novafitness SDS011 at 2 min resolution with 14 days of calibration followed by five months of validation in the field and obtained the best results for RLM, similarly to this study. As here, Cavaliere et al., is also one of the rare studies to have evaluated the performance of their calibration after the initial calibration period, in deployment conditions.

In the UK, the MCERTS for Indicative Ambient Particulate Monitors requires that a pair of PM instruments be tested for a time average of 24 h with an expanded uncertainty below 50% at a PM_2.5_ concentration of 25 μg/m^3^ and a between unit uncertainty <5 μg/m^3^. While the Performance Standards for this certification [4], specify that the time averaging could also be 1 h, it does not detail how the characterisation should be undertaken at such an averaging period. A total of 40 data pairs averaged over 24 h is required and the metrics should be evaluated for the full dataset and for the dataset split between concentration less than and greater than 18 μg/m^3^. Several recommendations can be made from the results obtained in this study.

1. Before calibration, out of the eight Sensirion SPS30 sensors tested, seven worked correctly and three met the 50% criteria for the expanded uncertainty for daily data (in most of cases tested), while the four other units did not consistently meet the criteria. Given the variability observed on the sensors tested here, randomly selected out of a batch 25 of Sensirion SPS30 before deployment, it is recommended to test more than two sensors of the same model and randomly select the sensors out of a batch of sensors. While this is easy to do for low-cost PM sensors, the certification is directed not only to low-cost PM sensors but also to instruments of higher cost that may not be produced in a batch and for which it may be too costly to test more than two units.
2. For the Plantower PMS5003, the expanded uncertainty obtained for hourly data was lower than the expanded uncertainty obtained for daily data with a narrower interquartile range for hourly data with no statistical differences between the different averaging windows. For the Sensirion SPS30, there were only marginal differences between the daily and the hourly data with the hourly data having a narrower interquartile range. This may be linked to the greater number of data points used for hourly data (40 for 24 h, 960 for 1 h and 28800 for 2 min averaging periods) than for 24 h data. This number of data point was chosen so that the sensors would be tested over 40 days regardless of the time averaging chosen to cover a wider range of environmental conditions, as emphasised in the Performance Standards (*4*). Given the observed use of PM sensors with hourly averages, and finer temporal resolutions, it is important in the future to find ways to certify them at a finer temporal granularity. However, the expanded uncertainty may not be appropriate to evaluate the sensors at temporal resolutions finer than daily readings. Indeed, the duration of 40 days for the certification should be upheld, ensuring the sensors are exposed to a broader range of PM concentrations and environmental conditions. The RMSE is proposed here as an alternative metric that is less sensitive to the number of sampling points and which proved to demonstrate substantial differences of performances between the different averaging windows tested. New performance standards would need to be developed for this metrics, and a value of 3 μg/m^3^ is proposed here based on the results obtained by the sensors with the calibration scenarios.
3. After a two-week calibration, the performances of the sensors exceeded the threshold required for the MCERTS for Indicative Ambient Particulate Monitors. The MCERTS for Indicative Ambient Particulate Monitors states that the sensors can be used for quantitative measurements once they have been calibrated through a two-week collocation with a reference instrument on the deployment site. The sensor must be re-calibrated every year or less. Considering the results obtained, it is strongly suggested that calibration should happen more frequently: preferably twice a year. Scenario 2w24w2w also enabled the performances to stay within the MCERTS for UK PM requirements, and this scenario is a solution to the shortcomings of the MCERTS for Indicative Ambient Particulate Monitors calibration frequency. This would mean at most two calibrations per year. The calibration frequency required (every year) should be revised to account for the performance variability observed here. The actual frequency necessary to maintain the performances of the sensors is a subject for further research, with twice per year being the maximum number of calibrations required with the calibration methods selected here. A longer study of at least two years would be required. One recommendation for the certification could be to require the sensors to be tested during at least two separate comparison campaigns during different seasons to account for seasonality in factors affecting reported data (beyond the actual airborne concentration of PM). This is reinforced by the results of previous studies regarding the specificity of the sensors to PM composition and size distribution [6].

Despite these issues, the results obtained in Fig. 8, Fig. 9 regarding the performances of the scenarios for daily averages, are strongly encouraging for the use of low-cost PM sensors and show that, with the proper calibration method and strategy, expanded uncertainty level below 25%, which is one of the requirements for MCERTS for Continuous Ambient Air Monitoring System [39] were reached, for the three subsets of data required. Here the best calibration methods were Multi-Linear Regression (MLR) and Robust Linear Model (RLM) based on the particle number concentrations reported by the sensors and RH. The best calibration strategy was calibrating the sensors before and after deployment, preferably for two weeks, although a one week calibration presented acceptable results for the shorter scenarios of 8 weeks of deployment. The certification is more stringent and does not allow for the sensors to be calibrated before and after their assessment. It also requires, amongst others, a laboratory evaluation of various parameters and that the sensors be tested during four comparison campaigns at a minimum of two different sites. Conditions on the slope and the intercept of the orthogonal least squares regression between the sensors and the reference method are also imposed. Scenarios 2w8w2w or 1w8w1w proposed here are most suitable for short-term measurement campaigns and scenarios 2w16w2w and 2w24w2w show that it is possible to use the sensors for longer-term measurements. Such scenarios would benefit sensor network architectures such as the one described in Cetinkaya *et al.* [46], where a combination of fixed and mobile low-cost sensors and fixed reference stations is envisioned. Scenario 2w24w2w obtained results that matched the Data Quality Objectives for indicative measurement, showing that calibrating the sensors twice a year enables them to reach performance in line with guidelines. Lower calibration frequencies may be sufficient but this would need a longer-term study over a duration of two years. This means that over a year, one mobile or transportable reference instrument, ideally with the same temporal resolution as the sensors, could be used to calibrate twelve locations with a rotation between the sites for two-weeks period. It is also possible that these calibrations could be done by using a gold-standard air quality monitor calibrated against a reference station and moved from site to site to calibrate other air quality monitors, further reducing the cost of maintaining the calibration of a network. However, future work is required about the “transportability” of the calibration methods developed here to facilitate the calibration of a network of sensors.

One element that could be further explored is the optimal number of particle number concentration size bins required to achieve good calibration results. Indeed, here, all the particle number concentration size bins of the Plantower PMS5003 were used for the multi-variate calibration models. This has implications for field deployments, if the six size bins were to be stored or transmitted during the deployment, which may be an issue when the bandwidth is limited and for which near-real-time data is important. In any case, these data can be stored locally on the device and collected later.

Scenarios 1w8w1w and 2w8w2w do not allow for real-time data as the data has to be corrected after the deployment. However, scenario 2w8w obtained results mostly below 50% of expanded uncertainty and the data could therefore be used directly with the caveat that they will be further correction later by adding the 1–2 weeks after deployment to the calibration. As a comparison, AURN data is ratified quarterly [47] while being available in real-time. The same could be applied to scenarios 2w16w and 2w24w. Hence, the calibration methods proposed here allow real-time data to be generated within the MCERTS for Indicative Ambient Particulate Monitors standards, and these data can be later improved to reach the 25% expanded uncertainty requirements of the MCERTS for UK PM standards, in a manner qualitatively similar to that which is done through post-validation of UK AURN station data. Coupled with these performances, the low calibration frequency required here, twice a year, is encouraging for the use of these low-cost PM sensors for complementing monitoring networks and providing useful data about personal-level exposure at time scales like those of bodies moving through a city and may help with source tracking.

## 5 Conclusion

The behaviour of the sensors was characterised at 10 s resolution, and a delay was observed between the sensors and the Fidas 200S. This delay can be linked to a range of environmental factor and to the difference of sampling line between the sensors and the Fidas 200S. In any case, given the low concentration levels generally measured by the sensors, the theoretical uncertainty leads to the choice of time averaging > ∼2 min and a time averaging of 2 min obtained a good compromise between increased performance and keeping a fine temporal resolution. Alternately, the limitations described to measure at a temporal resolution below 2 min linked to the total number of particles measured and hence to the sensing volume of the sensors and their airflow means that it is unlikely that smaller sensors will appear and the current size of the sensors will be a hard limit to the improvement of their performances. Additionally, if the levels of pollution decrease to levels complying with the WHO guideline of 5 μg/m^3^, sensor precision will be impacted, and this is also true for reference-grade instruments.

Different calibration methods were tested, with the indication that the performance of the calibrated sensors will vary over time, leading to the recommendation of whether to calibrate the sensors regularly (i.e. every two months or every six months) or calibrate the sensors before and after their deployment for short-term deployments.

The results revealed that the following additional challenges will arise for certification of the sensors.

• it is recommended to use more than two sensors and to select the sensors out of a batch randomly.
• if these sensors were to be certified at fine temporal resolution instead of daily resolution as is the current standard, the expanded uncertainty will not be suitable, and new metrics need to be used, such as the RMSE, with a limit value of 3 μg/m^3^, that are less dependent on the number of data points used in any comparison.
• the frequency of the calibration required (every year) should be revised to account for the performance variability observed here, with twice per year being the maximum number of calibrations required with the calibration methods selected here. Certification should require the sensors to be tested during at least two separate comparison campaigns during different seasons.

For both models of sensors, the calibration methods that obtained the best results were based on the particle number concentration reported by the sensors. This shows that it is preferable to use these measurements instead of the software calculated mass concentrations to limit the influence of potential built-in algorithms of the sensors. Calibration scenarios that calibrated the sensors before and after deployment obtained metrics that consistently passed the 50% expanded uncertainty threshold for indicative measurement and mainly around the 25% expanded uncertainty threshold for qualitative measurements required for reference instruments. This indicates that with the calibration methods and scenarios proposed here, the sensors can obtain similar performances as reference-grade instruments and provide valuable additional information for a wide range of applications for which spatio-temporal granularity is critical. These results were obtained at PM concentrations with a median value of 5.5 μg/m^3^ during the study. The data can be provided in real-time at 50% expanded uncertainty and improved a posteriori to 25% expanded uncertainty through a post-deployment calibration. Using the results of this study, one reference-grade instrument could be used to calibrate low-cost sensors in twelve different locations for the 2-weeks/24-weeks/2-weeks scenario rotating between the locations. It is also possible that these calibrations could be done by using a gold-standard air quality monitor calibrated against a reference station and moved from site to site to calibrate other air quality monitors, further reducing the cost of maintaining the calibration of a network. However, future work is required about the transportability of the calibration methods developed here to be facilitate the calibration of a network of sensors. The low frequency of calibration required here, of twice a year, is encouraging for the use of these low-cost PM sensors for complementing monitoring network and providing useful data about personal exposure at time scales similar to those of bodies moving through a city and help with source tracking.

## Funding sources

This work was supported by: 10.13039/501100000266Engineering and Physical Sciences Research Council UK grant EP/T517859/1 (FMJB).

Next Generation Unmanned Systems Science Centre for Doctoral Training supported by the 10.13039/501100000270Natural Environment Research Council UK grant NE/N012070/1 (FMJB).

Next Generation Unmanned Systems Science Centre Capital Grant 2018 and 2019 (FMJB, SO, GLF, SJC, ML, AKRM).

Leverhulme Trust through the Southampton Marine and Maritime Institute (FMJB)

Higher Education Innovation Funding (HEIF) from HEFCE to the Southampton Marine & Maritime Institute (SMMI), 10.13039/501100000739University of Southampton (SO, FMJB, PJB, NHCE, ML, AKRM, GLF, SJC).

Biotechnology and Biological Sciences Research Council Future Leader Fellowship grant BB/P011365/1 (ML).

National Institute for Health Research Southampton Biomedical Research Centre Senior Research Fellowship, and Biotechnology and Biological Research Council David Phillips Fellowship BB/V004573/1 (ML)

## Author contribution statement

Florentin M. J. Bulot: Conceived and designed the experiments; Performed the experiments; Analyzed and interpreted the data; Wrote the paper.

Steven J. Ossont: Andrew K. R. Morris: Simon J. Cox: Conceived and designed the experiments; Performed the experiments; Wrote the paper.

Philip J. Basford: Hazel L. Mitchell: Performed the experiments; Wrote the paper.

Natasha H. C. Easton: Contributed reagents, materials, analysis tools or data; Wrote the paper.

Gavin L. Foster: Matthew Loxham: Conceived and designed the experiments; Wrote the paper.

## Data availability statement

Data associated with this study has been deposited at the [https://doi.org/10.5281/zenodo.7198378].

## Additional information

Supplementary content related to this article has been published online at [URL].

## Declaration of competing interest

The authors declare that they have no known competing financial interests or personal relationships that could have appeared to influence the work reported in this paper.
