# Supplementary material for: Characterisation and calibration of low-cost PM sensors at high temporal resolution to reference-grade performance
Source: Heliyon. 2023 Apr 29;9(5):e15943. doi: 10.1016/j.heliyon.2023.e15943 (PMC10176080; doi:10.1016/j.heliyon.2023.e15943)
Supplement: 20230403_Supplementary materials [file mmc1.docx]

Supplementary materials

Characterisation and calibration of low-cost PM sensors at high temporal resolution to reference-grade performance

Florentin M. J. Bulot,^∗,1,2^ Steven J. Ossont,^3^ Andrew K. R. Morris,^4^ Philip J. Basford,^1^ Natasha H. C. Easton,^2,4,5,6^ Hazel L. Mitchell,^1^ Gavin L. Foster,^2,5,6^ Simon J. Cox,^1^ and Matthew Loxham^2,7,8,9^

**Affiliations**

^1^ Faculty of Engineering and Physical Sciences, University of Southampton, Southampton, UK

^2^ Southampton Marine and Maritime Institute, University of Southampton, Southampton, UK

^3^ BizData, 278 Collins St, Melbourne VIC 3000, AU

^4^ National Oceanography Centre, Southampton, UK

^5^ School of Ocean and Earth Science, National Oceanography Centre, University of Southampton, UK

^6^ Faculty of Environmental and Life Sciences, University of Southampton, Southampton, UK

^7^ School of Clinical and Experimental Sciences, Faculty of Medicine, University of Southampton, Southampton, UK

^8^ National Institute for Health Research Southampton Biomedical Research Centre, Southampton, UK

^9^ Institute for Life Sciences, University of Southampton, Southampton, UK

^*^ Corresponding author. Email: flo[rentin.bulot@centraliens.net](mailto:rentin.bulot@centraliens.net)

# Description of the Air Quality Monitors

The Air Quality Monitor (AQM), presented in Figure S1(B) is a modular air quality sensor platform which enables the deployment of multiple sensors, with data connectivity for remote administration and cloud‑based data storage. The enclosure measures 360 mm x 200 mm x 160 mm (H x W x D) and is weather-proof. The sensors are positioned with inlets facing downwards and the air flows through the enclosure through a grid of 8 mm diameter holes at the base of the enclosure and are protected from debris and biological material by a 3 mm diameter mesh. AQMs are controlled by a Raspberry Pi and are powered by Power over Ethernet. Each AQM includes five different models of PM sensors and a sensor for temperature and humidity (Sensirion SHT35 (*44*)). In this study, the focus was on two of these PM sensors: the Plantower PMS5003 and the Sensirion SPS30, presented in Figure S1(A).


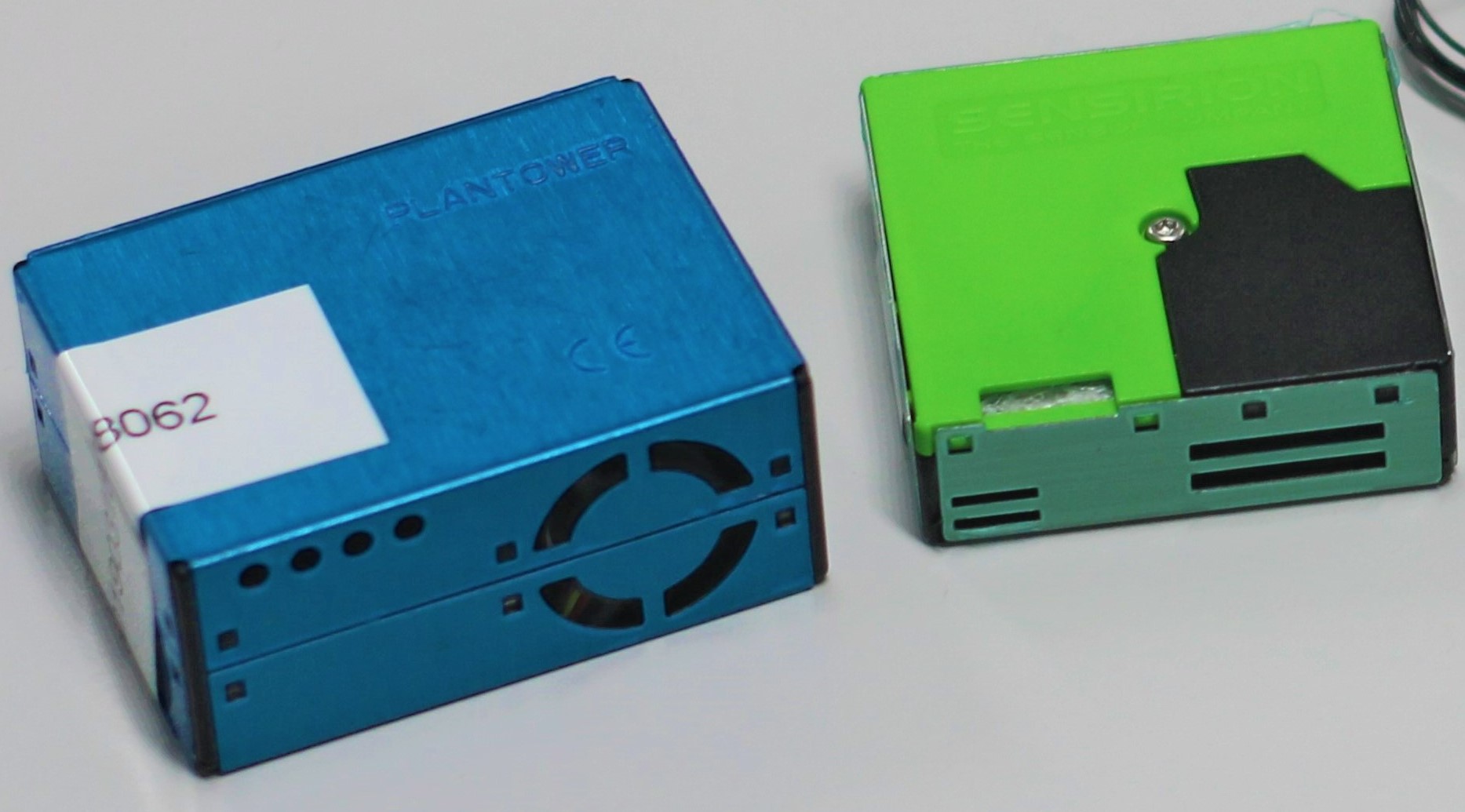
(**A**)


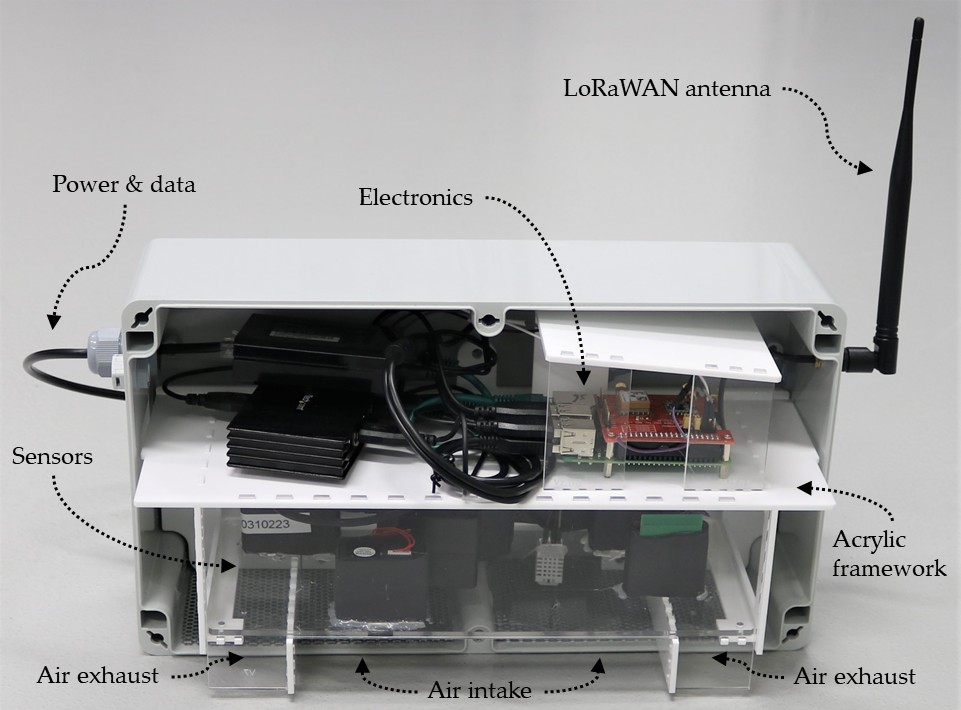


(**B**)

Fig S1. (A) Picture of the Plantower PMS5003 (left) and the Sensirion SPS30 (right), adapted from Bulot et al. (6). (B) Picture of the air quality monitors used in the study, adapted from Johnston et al. (*29*).

| SPS30 |  | n1 | n25 | n4 | n10 |
| --- | --- | --- | --- | --- | --- |
| R2 Equation | n05 | 0.995 0*.*87 *x* − 0*.*96 | 0.989 0*.*86 *x* − 1*.*27 | 0.985 0*.*86 *x* − 1*.*30 | 0.984 0*.*86 *x* − 1*.*31 |
| R2 Equation | n1 |  | 0.998 0*.*99 *x* − 0*.*53 | 0.998 0*.*99 *x* − 0*.*58 | 0.998 0*.*99 *x* − 0*.*59 |
| R2 Equation | n25 |  |  | 1 0*.*999 *x* − 0*.*07 | 1 0*.*999 *x* − 0*.*08 |
| R2 Equation | n4 |  |  |  | 1 0*.*999 *x* − 0*.*01 |

Table S1. Correlation and linear model between the different particle number concentration bin sizes reported by one of the Sensirion SPS30 during the period of the study. nXX represents the particle numbers between 0.3 and X.X µm. The equations are given as *y* = *a x* + *b* with *y* the left column and *x* the top row.

| PMS5003 |  | gr05um | gr10um | gr25um | gr50um | gr100um |
| --- | --- | --- | --- | --- | --- | --- |
| R2  Equation | gr03um | 0.982  4 *x* + 165 | 0.489  13 *x* + 490 | 0.491  144 *x* + 571 | 0.379  615 *x* + 647 | 0.279  1285 *x* + 755 |
| R2  Equation | gr05um |  | 0.680  4 *x* + 75 | 0.567  42 *x* + 101 | 0.427  177 *x* + 125 | 0.310  368 *x* + 157 |
| R2  Equation | gr10um |  |  | 0.865  11 *x* + 6 | 0.615  44 *x* + 13 | 0.435  90 *x* + 21 |
| R2  Equation | gr25um |  |  |  | 0.795  4 *x* + 0*.*5 | 0.574  9 *x* + 1 |
| R2  Equation | gr100um |  |  |  |  | 0.738  2 *x* + 0*.*2 |

Table S2. Correlation and linear model between the different particle number concentration bin sizes reported by one of the Plantower PMS5003 during the period of the study. grXXum represents the particle numbers greater than X.X µm (i.e. gr05um represents the particles greater than 0.5µm). The equations are given as *y* = *a x* + *b* with *y* the left column and *x* the top row.


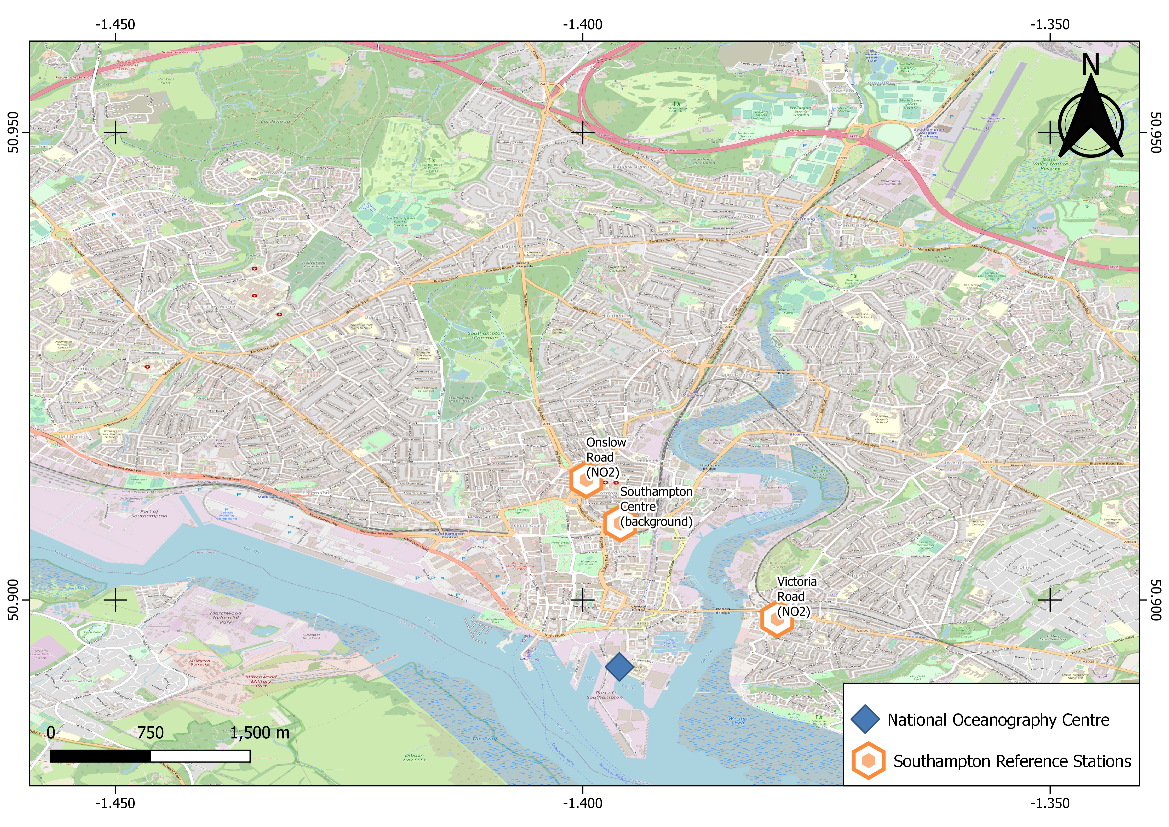


Fig S2. Map of Southampton, UK showing the National Oceanography Centre were the experiments took place and the AURN reference stations near in the city. Background map adapted from OpenStreetMap. The cartography in the OpenStreetMap map tiles is licensed under CC BY-SA (www.openstreetmap.org/copyright). The licence terms can be found on the following link: http://creativecommons.org/licenses/by-sa/2.0/.


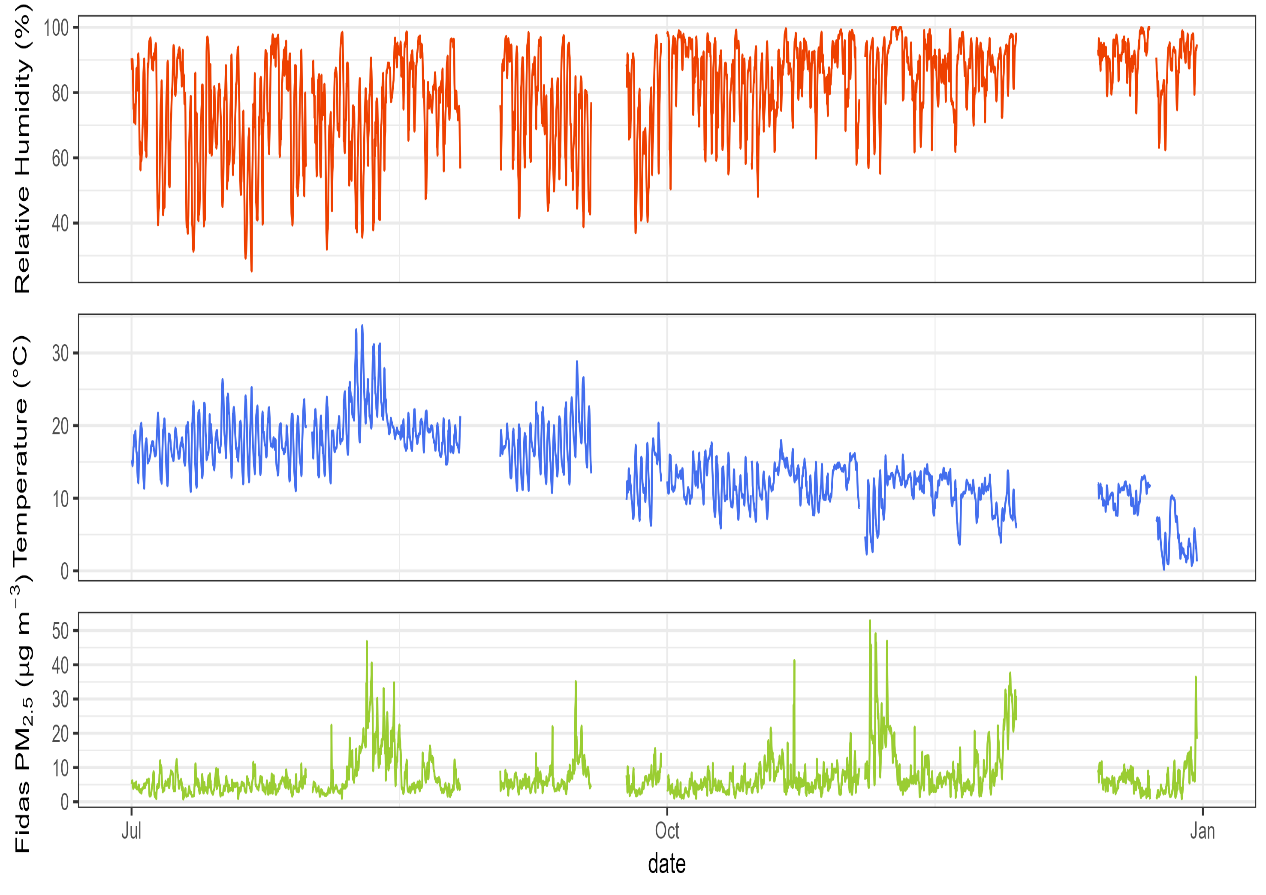


Fig S3. Time series of the 2 min averages for relative humidity, temperature and PM_2.5_ concentrations all measured by the Fidas 200S from 01/07/2020 until 31/12/2020 at the docks of the National Oceanography Center in Southampton, UK.


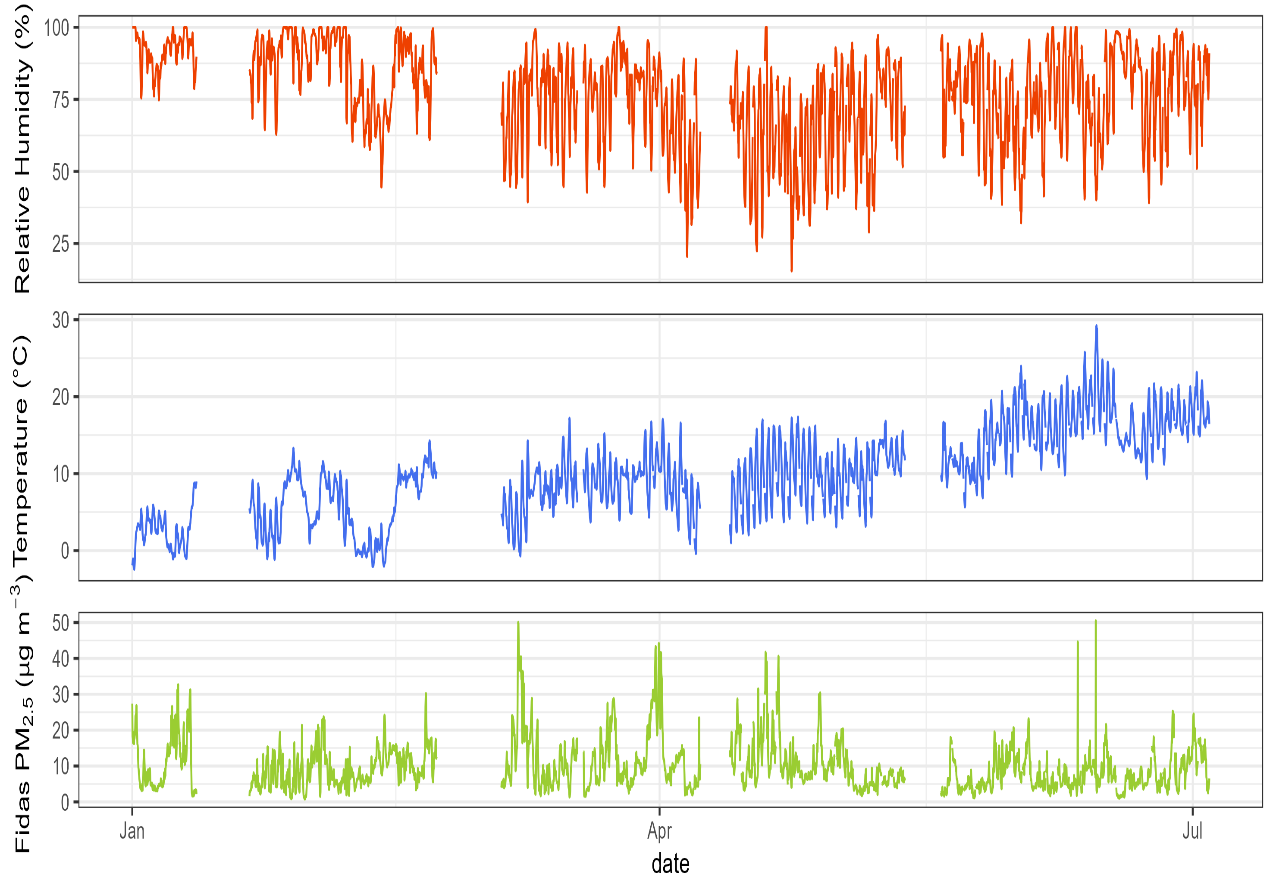


Fig S4. Time series of the 2 min averages for relative humidity, temperature and PM_2.5_ concentrations all measured by the Fidas 200S from 01/01/2021 until 03/07/2021 at the docks of the National Oceanography Center in Southampton, UK.


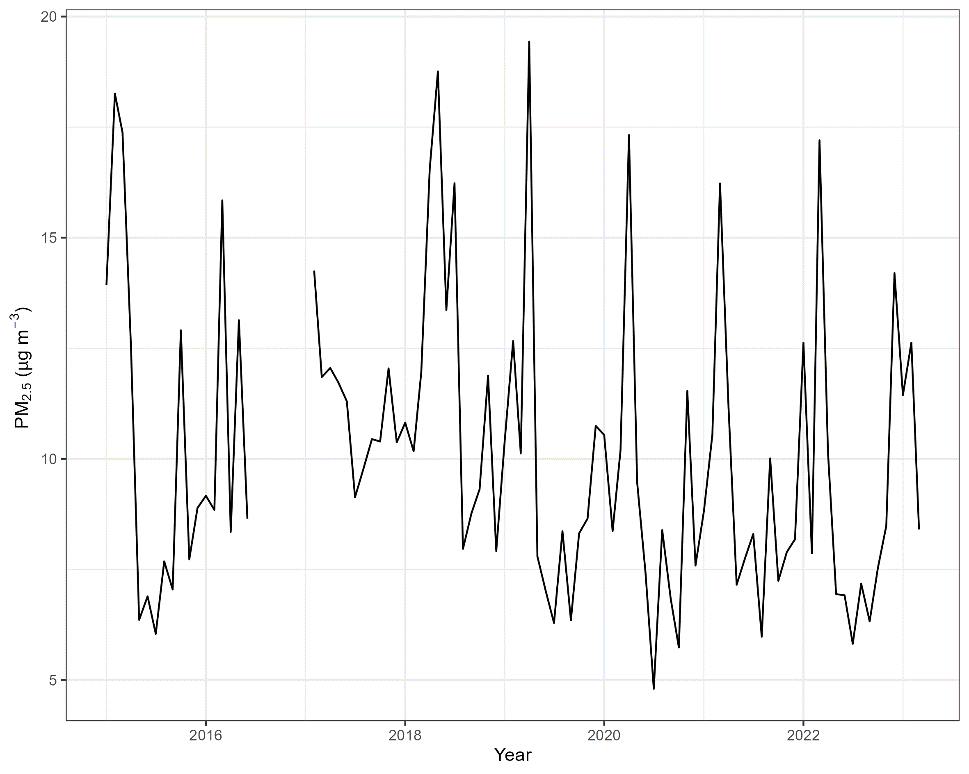


Fig S5. Time series of the monthly PM_2.5_ concentration measured by the AURN station Southampton Centre from 2015 to 2023.

| Method | Variable | Second step |
| --- | --- | --- |
| LM | PM2*.*5 | - |
| OLS | PM2*.*5 | - |
| MLR | PM_2_*_._*_5_, RH | - |
|  | PM*number* | - |
|  | PM*number* , RH | - |
| *κ*-koehler | PM_2_*_._*_5_, RH | OLS/LM |
|  | PM*number* ,RH | OLS/LM |
| RLM | PM2*.*5 | - |
|  | PM_2_*_._*_5_ RH | - |
|  | PM*number* , RH | - |
| SVM | PM2*.*5 | - |
|  | PM_2_*_._*_5_, RH | - |
| GBM | PM2*.*5 | - |
|  | PM_2_*_._*_5_, RH | - |

Table S3. Calibration methods and input variables used. Linear Model (LM), Orthogonal Least Square (OLS), Multi-Linear Regression (MLR), *κ*-koehler, Robust Linear Model (RLM), Support Vector Machines (SVM) and Gradient Boosting Machines (GBM).

# Examples of subsets used for the data analysis

## 1w8w1w

The first subset is composed of the following days (yyyy-mm-dd date format):

2020-07-01; 2020-07-02; 2020-07-03; 2020-07-04; 2020-07-05; 2020-07-06; 2020-07-07; 2020-07-08; 2020-07-09; 2020-07-10; 2020-07-11; 2020-07-12; 2020-07-13; 2020-07-14; 2020-07-15; 2020-07-16; 2020-07-17; 2020-07-18; 2020-07-19; 2020-07-20; 2020-07-21; 2020-07-22; 2020-07-23; 2020-07-24; 2020-07-25; 2020-07-26; 2020-07-27; 2020-07-28; 2020-07-29; 2020-07-30; 2020-08-01; 2020-08-02; 2020-08-03; 2020-08-04; 2020-08-05; 2020-08-06; 2020-08-07; 2020-08-08; 2020-08-09; 2020-08-10; 2020-08-11; 2020-08-12; 2020-08-13; 2020-08-14; 2020-08-15; 2020-08-16; 2020-08-17; 2020-08-18; 2020-08-19; 2020-08-20; 2020-08-21; 2020-08-22; 2020-08-23; 2020-08-24; 2020-08-25; 2020-08-26; 2020-09-02; 2020-09-03; 2020-09-04; 2020-09-05; 2020-09-06; 2020-09-07; 2020-09-08; 2020-09-09; 2020-09-10; 2020-09-11; 2020-09-12; 2020-09-13; 2020-09-14; 2020-09-15

And the second subset is composed of the following days:

2020-07-02; 2020-07-02; 2020-07-03; 2020-07-04; 2020-07-05; 2020-07-06; 2020-07-07; 2020-07-08; 2020-07-09; 2020-07-10; 2020-07-11; 2020-07-12; 2020-07-13; 2020-07-14; 2020-07-15; 2020-07-16; 2020-07-17; 2020-07-18; 2020-07-19; 2020-07-20; 2020-07-21; 2020-07-22; 2020-07-23; 2020-07-24; 2020-07-25; 2020-07-26; 2020-07-27; 2020-07-28; 2020-07-29; 2020-07-30; 2020-08-01; 2020-08-02; 2020-08-03; 2020-08-04; 2020-08-05; 2020-08-06; 2020-08-07; 2020-08-08; 2020-08-09; 2020-08-10; 2020-08-11; 2020-08-12; 2020-08-13; 2020-08-14; 2020-08-15; 2020-08-16; 2020-08-17; 2020-08-18; 2020-08-19; 2020-08-20; 2020-08-21; 2020-08-22; 2020-08-23; 2020-08-24; 2020-08-25; 2020-08-26; 2020-09-02; 2020-09-03; 2020-09-04; 2020-09-05; 2020-09-06; 2020-09-07; 2020-09-08; 2020-09-09; 2020-09-10; 2020-09-11; 2020-09-12; 2020-09-13; 2020-09-14; 2020-09-16

# Preliminary method selection

## Additional calibration methods considered

Two additional calibration methods have been tested during the preliminary method selection presented in here: Support Vector Machines (SVM) and Gradient Boosting Machines (GBM). SVM is a non-linear statistical learning method using kernels (*45*). It was implemented for PM mass concentration and RH using the package "e1071" (*46*) in R. The SVM used a radial kernel, with a cost of 10 and a *γ* = 10*^−^*^2^. GBM is a non-linear statistical learning method based on weighted decision trees (*47*). The GBM parameters used here were 1000 trees, a shrinkage of 0.1, an interaction depth of 6 and minimum number of observations in the terminal nodes of the trees of 10 and a 10-fold cross validation. It was implemented using the "gbm" package (*48*) in R. The parameters of SVM and GBM were chosen based on the parameters generally recommended for these methods. The parameters described above should be tuned and optimised but the goal of this study was to use out-of-the box calibration techniques and these methods already require more compute power than the other methods presented.

## Preliminary method selection

In this section, the sensors were calibrated using the first two weeks of data collected and the next 40 days to evaluate the calibration, as detailed in the methods. Figure S4 presents the Root Mean Square Error (RMSE) obtained by the two models of sensors for the different calibration methods considered on the training and the evaluation datasets. The GBM models, trained using a 10-fold cross validation, obtained virtually perfect scores on the training dataset, which likely indicates over fitting, and did not achieve the same results on the evaluation dataset. This is likely due to the fact that the parameters of the models have not been tuned. Additionally, to perform well, these models need to capture a wide range of conditions for their predictors (here PM concentrations and RH) which is not really feasible in most cases during a short field calibration. It would be impractical for longer calibration studies focused on 2 min data given the computational overhead of these machine learning techniques. These methods led here to clipping and inappropriate correction as can be seen in Supplementary Figure S5. This clipping can also be observed for the Support Vector Machines (SVM) models. Due to the negative results obtained for these two methods resulted in them being excluded from further analysis, although optimisation and tuning of the hyper-parameters of the two models would be required to fully determine whether these models are useful for our current purpose.

For the Plantower PMS5003, compared to the situation without calibration, all the models used reduced the RMSE and the between unit uncertainty of the sensors and reduced the interquartile range of the two metrics. The best results for the RMSE were obtained by the model based on Koehler mass correction with Orthogonal Least Square (OLS). In contrast, for the Sensirion SPS30, compared to the initial situation, the methods based on Laulainen RH correction with Linear Regression based on ordinary least square (LR) increased the RMSE and the methods based on Laulainen and Koehler correction increased the between unit uncertainty.


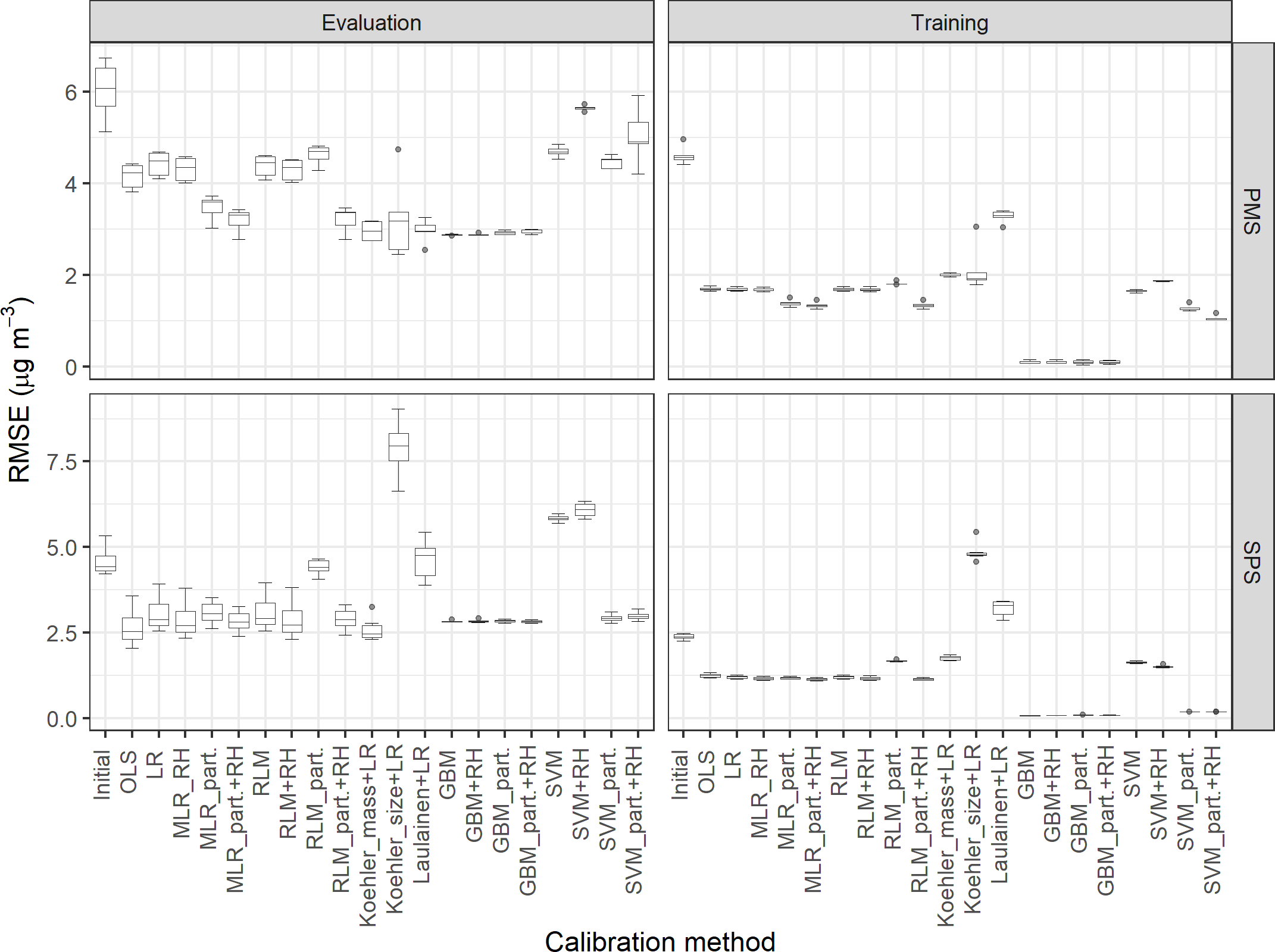


Fig S6. Box and Whisker plot of the Root Mean Square Error (RMSE) obtained by the two models of sensors using different calibration methods on a training dataset and on a calibration dataset. The Box and Whisker corresponds to each individual sensor of the model considered. The Box and Whisker plot horizontal lines represent, from bottom to top, the lower quartile, the median and the upper quartile. The vertical lines are drawn to the smallest and the largest data point that fall within 1.5 times the interquartile range below the lower quartile and above the upper quartile respectively. The dots are data points outside of that range considered as outliers.


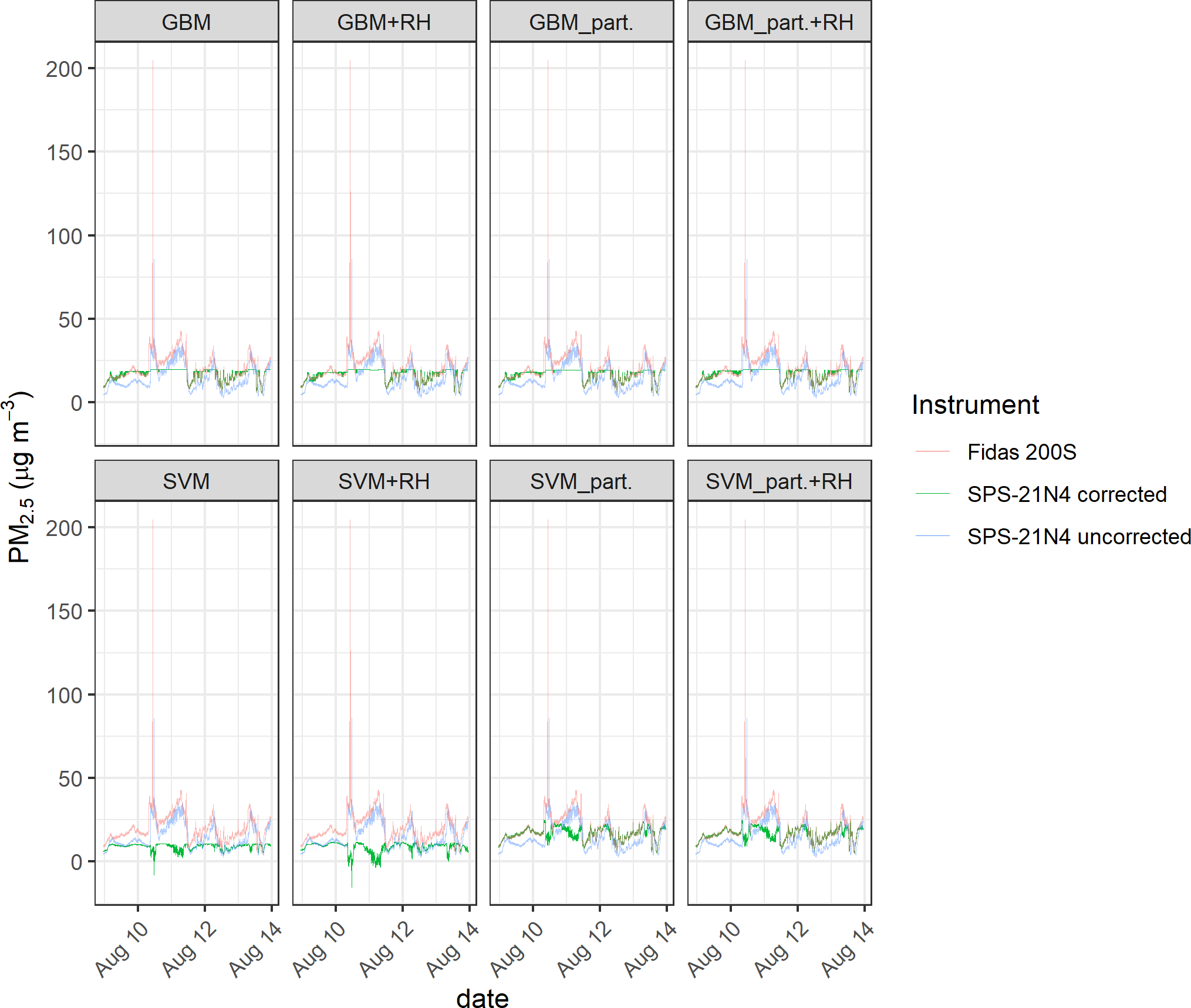


Fig S7. PM_2.5_ measurements of the Sensirion SPS-21N4 before and after correction with Gradient Boosting Machines (GBM), GBM with Relative Humidity (GBM+RH), GBM on particle number concentration (GBM_part.), GBM on particle number concentration with Relative Humidity (GBM_part.+RH), Support Vector Machines (SVM), SVM with Relative Humidity (SVM+RH), SVM on particle number concentration (SVM_part.), SVM on particle number concentration with Relative Humidity (SVM_part.+RH) and comparison with the measurements of the Fidas 200S between 09/08/2020 and 14/08/2020.


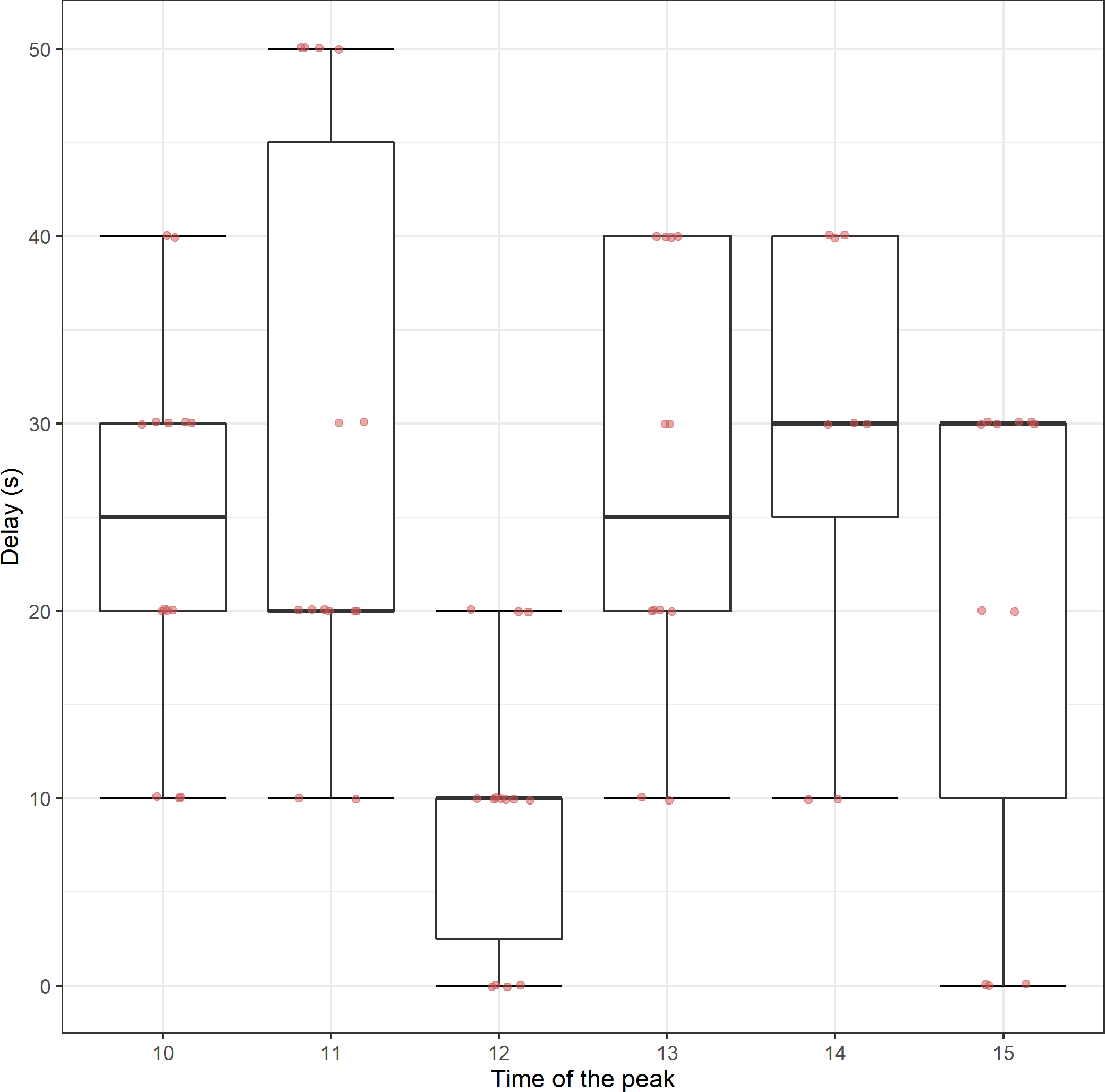


Fig S8. Delay observed between the sensors and the Fidas during the incense experiment. The Box and Whisker plot horizontal lines represent, from bottom to top, the lower quartile, the median and the upper quartile. The vertical lines are drawn to the smallest and the largest data point that fall within 1.5 times the interquartile range below the lower quartile and above the upper quartile respectively. The actual data points are drawn in red.

| Calibration method | Mean | Median | 25% quantile | 75% quantile | Interquartile range |
| --- | --- | --- | --- | --- | --- |
|  | (µg*/*m^3^) | (µg*/*m^3^) | (µg*/*m^3^) | (µg*/*m^3^) | (µg*/*m^3^) |
| Initial | 6.6 | 6.8 | 5.6 | 7.5 | 1.8 |
| OLS | 4.0 | 3.8 | 3.1 | 4.4 | 1.3 |
| LR | 3.9 | 3.8 | 3.2 | 4.4 | 1.2 |
| MLR_RH | 4.2 | 3.8 | 3.2 | 4.4 | 1.2 |
| MLR_part. | 3.4 | **3.3** | **2.6** | **3.7** | **1.1** |
| MLR_part.+RH | 3.5 | **3.2** | **2.6** | **3.7** | **1.2** |
| RLM | 3.8 | 3.6 | 3.0 | 4.2 | 1.2 |
| RLM+RH | 4.0 | 3.7 | 3.0 | 4.2 | 1.2 |
| RLM_part. | 3.6 | 3.4 | 2.9 | 3.9 | 1.0 |
| RLM_part.+RH | 3.4 | **3.1** | **2.6** | **3.7** | **1.1** |
| Koehler_mass+LR | 5.6 | 5.2 | 3.5 | 6.7 | 3.2 |
| Koehler_size+LR | 5.0 | 4.1 | 3.4 | 5.3 | 1.9 |
| Laulainen+LR | 9.1 | 6.6 | 4.3 | 11.5 | 7.2 |

Table S4. Summary for the Root Mean Square Error (RMSE) for the Plantower PMS5003 on the evaluation period for the different calibration methods considered for all the combinations of two weeks of calibrations followed by 40 days of evaluation during the study. The statistics are summarised for every sensor of the model.

| Calibration method | Mean | Median | 25% quantile | 75% quantile | Interquartile range |
| --- | --- | --- | --- | --- | --- |
|  | (µg*/*m^3^) | (µg*/*m^3^) | (µg*/*m^3^) | (µg*/*m^3^) | (µg*/*m^3^) |
| Initial | 3.9 | 3.8 | 3.2 | 4.6 | 1.3 |
| OLS | 3.9 | 3.8 | 2.9 | 4.4 | 1.5 |
| LR | 3.5 | 3.4 | 2.8 | 4.0 | 1.3 |
| MLR_RH | 3.8 | 3.4 | 2.7 | 4.1 | 1.4 |
| MLR_part. | 3.4 | **3.3** | **2.6** | **3.8** | **1.2** |
| MLR_part.+RH | 3.6 | **3.2** | **2.6** | **3.9** | **1.3** |
| RLM | 3.4 | 3.3 | 2.7 | 4.0 | 1.3 |
| RLM+RH | 3.7 | 3.3 | 2.6 | 4.0 | 1.4 |
| RLM_part. | 3.6 | 3.5 | 2.6 | 4.2 | 1.6 |
| RLM_part.+RH | 3.3 | **3.0** | **2.5** | **3.8** | **1.3** |
| Koehler_mass+LR | 5.7 | 4.9 | 3.8 | 7.0 | 3.2 |
| Koehler_size+LR | 12.7 | 10.8 | 7.9 | 17.2 | 9.3 |
| Laulainen+LR | 9.9 | 7.0 | 5.3 | 13.8 | 8.5 |

Table S5. Summary for the Root Mean Square Error (RMSE) for the Sensirion SPS30 on the evaluation period for the different calibration methods considered for all the combinations of two weeks of calibrations followed by 40 days of evaluation during the study. The statistics are summarised for every sensor of the model.

Sensor model Calibration method 25*^th^* quartile Median 75*^th^* quartile

(%) (%) (%)

Plantower PMS5003 Initial 95 116 145

MLR part. 12 20 30

MLR part.+RH 12 18 29

RLM part. 12 18 29

RLM part.+RH 11 17 27

Sensirion SPS30 Initial 29 41 53

MLR part. 14 22 32

MLR part.+RH 14 21 30

RLM part. 13 20 30

RLM part.+RH 13 20 28

Table S6. Expanded uncertainties obtained for the full dataset, with daily averages, by the Plantower PMS5003 and Sensirion SPS30 for the calibration scenario 2w8w2w for Multi-Linear Regression (MLR) and Robust Linear Model (RLM) on particle number and with and without Relative Humidity (RH).
